# Alzheimer's disease‐induced phagocytic microglia express a specific profile of coding and non‐coding RNAs

**DOI:** 10.1002/alz.13502

**Published:** 2023-10-12

**Authors:** Flavia Scoyni, Luca Giudice, Mari‐Anna Väänänen, Nicholas Downes, Paula Korhonen, Xin Yi Choo, Nelli‐Noora Välimäki, Petri Mäkinen, Nea Korvenlaita, Annemieke J Rozemuller, Helga E de Vries, Jose Polo, Tiia A Turunen, Seppo Ylä‐Herttuala, Thomas B Hansen, Alexandra Grubman, Minna U Kaikkonen, Tarja Malm

**Affiliations:** ^1^ A.I.Virtanen Institute for Molecular Sciences University of Eastern Finland Kuopio Finland; ^2^ Department of Anatomy and Developmental Biology Monash University Clayton Australia; ^3^ Development and Stem Cells Program Monash Biomedicine Discovery Institute Clayton Victoria Australia; ^4^ Australian Regenerative Medicine Institute Monash University Clayton Victoria Australia; ^5^ Department of Pathology, Amsterdam UMC, Vrije Universiteit Amsterdam VU University Medical Center Amsterdam MB the Netherlands; ^6^ Department of Molecular Cell Biology and Immunology, Amsterdam UMC, Vrije Universiteit Amsterdam VU University Medical Center Amsterdam MB the Netherlands; ^7^ Interdisciplinary Nanoscience Center Department of Molecular Biology and Genetics Aarhus University Aarhus Denmark; ^8^ Present address: Targovax ASA Lysaker 1366 Norway

**Keywords:** Alzheimer's disease, microRNAs, microglia, phagocytosis, post‐transcriptional regulation

## Abstract

**INTRODUCTION:**

Alzheimer's disease (AD) is a neurodegenerative disease and the main cause of dementia in the elderly. AD pathology is characterized by accumulation of microglia around the beta‐amyloid (Aβ) plaques which assumes disease‐specific transcriptional signatures, as for the disease‐associated microglia (DAM). However, the regulators of microglial phagocytosis are still unknown.

**METHODS:**

We isolated Aβ‐laden microglia from the brain of 5xFAD mice for RNA sequencing to characterize the transcriptional signature in phagocytic microglia and to identify the key non‐coding RNAs capable of regulating microglial phagocytosis. Through spatial sequencing, we show the transcriptional changes of microglia in the AD mouse brain in relation to Aβ proximity.

**RESULTS:**

Finally, we show that phagocytic messenger RNAs are regulated by *miR‐7a‐5p, miR‐29a‐3p* and *miR‐146a‐5p* microRNAs and segregate the DAM population into phagocytic and non‐phagocytic states.

**DISCUSSION:**

Our study pinpoints key regulators of microglial Aβ clearing capacity suggesting new targets for future therapeutic approaches.

## 1 BACKGROUND

Microglia serve as the main immune effector cells of the brain, acting as the first line of defense against any invading pathogens.^1^, ^2^ Under healthy conditions, microglia exert essential homeostatic and neuron‐supportive functions. However, in neurodegenerative diseases, such as Alzheimer's disease (AD), these critical activities become impaired.^2^ Among the elderly, AD is the most common cause of dementia^3^ and synapse loss with concomitant accumulation of intraneuronal tau tangles and extracellular beta‐amyloid (Aβ) plaques.^4^, ^5^ In addition, these abnormally accumulated protein aggregates are accompanied by microglial activation. Numerous Genome‐Wide Association Studies have highlighted the role of microglia in AD, identifying risk loci in genes highly or specifically expressed in microglia,^6^, ^7^, ^8^ suggesting that microglial dysfunction might contribute to the accumulation and inadequate clearance of Aβ during the disease pathogenesis. Instead, microglia acquire disease‐related states in the AD brain and show specific transcriptional signatures.^9^, ^10^ The transcriptional signature of microglia capable of phagocytosing Aβ in the brains of 5xFAD mice^11^ shows dysregulation in the expression of gene related to immune, metabolic, and phagocytic processes that can be further induced by exposure to Aβ‐containing environment. Single‐cell sequencing studies of the brain of the 5xFAD mice have pinpointed a specific disease‐associated microglia (DAM) state.^9^ The accumulation of Aβ and tau hyperphosphorylated proteins, coupled with impaired aggregate clearance, triggers cellular oxidative stress and sustained neuroinflammation in microglia.^4^, ^5^ A link between induced phagocytosis of Aβ and synapse loss is evident from the findings showing that hypoxia‐inducible factor‐1α (HIF‐1α), transcription factor essential in cellular adaptation during oxidative stress,^12^ is prominently upregulated in Aβ‐containing microglia in 5xFAD mouse brain, and is also associated with synapse loss in AD.^13^ While the connection between Aβ‐induced phagocytosis and synapse loss has been established, the molecular regulators responsible for microglial phagocytosis in the AD brain remain unknown.

One potential class of regulators of microglial phagocytosis is microRNAs (miRNAs), which are short (∼22 nucleotides long) non‐coding RNAs capable to post‐transcriptionally regulate the expression of messenger RNAs (mRNAs) leading to their translational repression or promoting their degradation.^14^ Previous studies have demonstrated the dysregulation of miRNA expression in bulk RNA sequencing from AD brain tissue^15^ and have pinpointed miRNAs capable of altering microglial activities.^16^ However, the specific miRNAs responsible for converting microglia to a phagocytic phenotype in the AD brain in vivo are largely unexplored. Investigating and understanding these specific miRNAs could reveal potential targets for therapeutic interventions, ultimately aimed at enhancing microglial phagocytosis in AD.

Here, we aim to discover key microglial miRNAs which functionally regulate phagocytosis and drive specific microglial phenotypes in AD. Identification of these miRNAs holds great relevance for the development of novel therapeutics, shedding light on the molecular mechanisms underlying microglial dysfunction, neuroinflammation, and clearance deficits observed in AD. Manipulating these miRNAs could potentially guide microglial function and enhance the clearance of pathological protein aggregates, presenting promising avenues for the development of targeted and precision medicine approaches to treat AD.

**RESEARCH IN CONTEXT**

1. **Systematic review**: Alzheimer's disease (AD) is the first cause of dementia in the elderly marked by the accumulation of microglia cells around beta‐amyloid (Aβ) clusters in the brain. Recent research identified unique AD‐related genes in microglia, but the understanding of molecular effectors, including coding and non‐coding RNAs, driving the phagocytic state remains limited.
2. **Interpretation**: Our study, pinpointed key microRNAs (miRNAs) regulating messenger RNAs (mRNAs) responsible for controlling microglial phagocytic activity. These mRNAs proved to be indicators of a distinct microglial subtype linked to AD. Through an integrative approach, we show that miRNAs play a crucial role in defining microglial phagocytic state enhancing the comprehension of AD pathophysiology.
3. **Future directions**: As RNA molecules have enormous potential for therapeutic interventions, we hope that our work could bridge technical characterization and development of more effective therapeutic approaches for a wide range of diseases.

## 2 METHODS

### 2.1 Animal housing and procedures

5xFAD animals were bred in the Laboratory animal center of the University of Eastern Finland under the license approved by Regional State Administrative Agency of Finland (ESAVI‐2021‐002938) and Monash Animal Ethics Committee (MARP/2016/112) and conformed to national and institutional guidelines. In this study, a total of nine wild‐type and nine 5xFAD transgenic animals were used at the age of 5 and 5.5 months. Animals were housed in individually ventilated cages with unlimited food and water supply and 12 h light‐dark cycle. The animals were genotyped from ear samples. DNA was extracted by heating the samples to 95°C in 50 mM NaOH. pH was set after heating using 1 M Tris‐HCl, pH 8.0. Genotyping was done using primers for *Psen1* (Integrated DNA Technologies, Inc., US) and DreamTaq Green DNA Polymerase (ThermoFisher Scientific, US). After polymerase chain reaction (PCR), the samples were run on agarose gel and imaged using ChemiDoc imaging device (BioRad Laboratories, Inc., US).

### 2.2 BV‐2 cell culture and treatments

Murine glioblastoma cell line (BV‐2) cells were cultured in RPMI 1640 Medium, GlutaMAX™ Supplemented base medium (Gibco, 31966021), 1% penicillin‐streptomycin and 10% heat‐inactivated and filtered FBS (Gibco, 10270106) in temperature controlled humidified incubator (37 °C, 5% CO2). One day prior transfection, cells were seeded in six‐well plates (120.000 cells/ well), then cells were transfected with Viromer® BLUE (Lipocalyx GmbH, VB‐01LB‐00) using 20 nM of miRIDIAN scramble mimic (cat: CN‐002000‐01‐05, Dharmacon™), mirna mimic (mmu‐miR‐744‐5p cat: C‐310776‐03‐0002; mmu‐miR‐532‐5p cat: C‐310769‐01‐0002; mmu‐miR‐339‐5p cat: C‐310548‐07‐0002; mmu‐miR‐148b‐3p cat: C‐310546‐05‐0002; mmu‐miR‐7a‐5p mimic cat: C‐310591‐07‐0002; mmu‐miR‐29a‐3p mimic cat: C‐310521‐07‐0002; mmu‐miR‐146a‐5p mimic cat: C‐310423‐05‐0002; mmu‐miR‐140‐3p cat: C‐310417‐07‐0002; mmu‐let‐7 g‐5p cat: C‐310374‐07‐0002 Dharmacon™), or positive control targeting glyceraldehyde 3‐phosphate dehydrogenase (GAPDH; cat: CP‐002000‐02‐05, Dharmacon™) or 100 nM of miRCURY LNA miRNA Inhibitor (mmu‐miR‐7a‐5p cat: YI04100818‐ADA, mmu‐miR‐29a‐3p cat: YI04100172‐ADA, mmu‐miR‐146a‐5p cat: YI04100680‐ADA, negative control A cat: YI00199006‐ADB, Qiagen) following manufacturer protocol for 6 h and then the media was changed to fresh media. To define the best mimic concentration efficiency cells were transfected with 5, 10, 20, 35, and 50 nM of scramble mimic or positive control targeting GAPDH and after incubation with pHrodo™ Green *E. coli* BioParticles™ (Invitrogen, P35366) phagocytosis beads for 20 min, cells were stained for viability with LIVE/DEAD™ Fixable Aqua Dead Cell Stain Kit (Invitrogen, L34957) following the manufacturer protocol. To define the optimal concentration for efficient transduction of miRNA inhibitors, the cells were transfected with 25, 50, 75, and 100 nM of FAM scramble inhibitor and then stained for viability with LIVE/DEAD™ Fixable Aqua Dead Cell Stain Kit (Invitrogen, L34957) following the manufacturer protocol. Viability and phagocytic activity upon transfection were detected with Cytoflex S (Beckman Coulter) and analyzed with FlowJo™ Software v10.8 Software (Becton, Dickinson and Company; 2023) (Supplementary Table S1). The concentration with highest viability and unaffected changes in phagocytosis by scramble mimic of 20 nM was validated for efficiency through RNA extraction and qPCR of the positive control targeting GAPDH (Supplementary Figure S1). The concentration of 100 nM of miRNA inhibitor was chosen for the experiment. In this experiment, lipopolysaccharide (LPS) treatment was included, and a concentration of 10 ng/ml was chosen for the treatment experiments.

### 2.3 Phagocytosis assay

Upon transfection in 6‐well plates, cells were detached and plated on 96 well plates pre‐coated with 1× PDL at a density of 8000 cells/well. After induction for 24 h in LPS 10 ng/ml or vehicle, pHrodo™ Green E. coli BioParticles™ (Invitrogen, P35366) phagocytosis beads were resuspended in 10 ml Optimem media per vial following the manufacturer protocol and added in the volume of 50 μl per well. BV‐2 cells were imaged every 30 min for a total of 6 h with IncuCyte® S3 Live Cell Analysis System (Essen BioScience Ltd.) in bright field and green channel live cell images (two 10× magnification images per well). Before adding the beads, images in bright field were taken to detect the confluency of each well and immediately after adding the beads in the green channels to detect the fluorescence background. Analysis of the phagocytosis assay was performed on background subtracted fluorescence value normalized on confluence with an average of the six technical replicates.

### 2.4 Microglia cells FACS isolation

As before,^10^ 2 h before sacrifice, mice were intraperitoneally injected with methoxy‐X04 5 mg/kg (2 mg/ml dissolved 1 to 1 into dimethyl sulfoxide (DMSO) and resuspended 0.9% saline solution). After intracardial perfusion, microglia was isolated by mechanical dissociation and density gradient. The density gradient was generated resuspending the cell pellet in 70% Percoll in PBS overlayed with 37% Percoll solution in PBS and centrifuged at 2000 g for 20 min in a refrigerated centrifuge. Microglia was obtained by the 37‐70% Percoll gradient and washed 1:5 in ice‐cold PBS and spun at maximum speed in refrigerated microcentrifuge for 1 min. Microglia cells were isolated with FACSAria™ III cell sorter (BD Biosciences) after staining with CD11b‐BV650 (1:200 Biolegend, 141723), CD45‐BV786 (1:200, BD Biosciences 564225), and CX3CR1‐FITC (1:100, Biolegend, 149019). Microglia were gated as live (propidium iodide negative), CD11b+, CD45+, CX3CR1+ single cells, and Me‐X04^+^ and Me‐X04^−^ microglial populations in transgenic 5xFAD mice were sorted separately for further analysis (Supplementary Figure S2). The flow cytometry results were analyzed using FlowJo™ Software v10.8 Software (Becton, Dickinson and Company; 2023).

### 2.5 RNA extraction

RNA from sorted microglia from 5xFAD mice injected with Me‐X04 was extracted with Qiagen Micro RNA kit. Mimic transfected BV‐2 cells RNA for RNA sequencing, quality control and the *post mortem* human brain tissue RNA were extracted using TRIzol™ Reagent (Invitrogen). All the RNA extractions were performed following the manufacturer's instructions.

### 2.6 qPCR

RNA extracted from *post mortem* human brain tissues was reverse‐transcribed with TaqMan™ MicroRNA Reverse Transcription Kit (Invitrogen) using miRNA‐specific primers supplied in the TaqMan® probe kit for qPCR (hsa‐miR‐7‐5p Assay ID: 005723_mat; hsa‐miR‐29a‐3p Assay ID: 002112; has‐miR‐146a‐5p Assay ID: 000468; U6 snRNA Assay ID: 001973) following the manufacturer's protocol. The qPCR was performed using Maxima Probe/ROX qPCR Master Mix (Invitrogen) and TaqMan® miRNA‐specific probes (Thermofisher) following the manufacturer's protocol. The result was analyzed with the ΔΔCT method and normalized to U6 snRNA.

### 2.7 Library preparation

Total RNA was isolated using Qiagen Micro RNA kit or TRIzol™ Reagent as specified in the RNA extraction section, and the yield and integrity (RIN > 8) for each sample was assessed using the Agilent 2100 Bioanalyzer (Agilent Technologies, US), respectively. Total RNA from sorted microglia from 5xFAD mice injected with Me‐X04 was used to construct RNA‐seq libraries using the Ovation RNA‐Seq System V2 followed by Ovation Ultralow library System V2 (Tecan Trading AG, Switzerland). For miRNA sequencing from the same type of samples, the QIAseq miRNA Library Kit (Qiagen, Germany) was used to generate the libraries. For RNA sequencing of BV‐2 cells transfected with miRNA mimic and LPS treatment, we used CORALL total RNA‐Seq Library Prep Kit (Lexogen GmbH, Austria) after ribosomal RNA depletion with RiboCop rRNA Depletion Kit V1.2 (Lexogen GmbH, Austria). All the libraries were generated following the manufacturer's protocol.

All the libraries were size profiled using the Agilent 2100 Bioanalyzer (Agilent Technologies, US) and quantitated with Qubit™ High Sensitivity DNA kit (Invitrogen). Transcripts from sorted microglia from 5XFAD mice were sequenced on the HiSeq™ 3000 platform (Illumina, USA) using 50 cycle single end reads. Additional samples were later sequenced using the NextSeq™ 500 platform (Illumina, US) using 75 cycle end reads. Small RNA‐seq libraries were sequenced using the NextSeq™ 500 platform (Illumina, US) using single end 75 cycles. BV‐2 mirna transfected samples were sequenced single end 75 cycles on NextSeq™ 550 (Illumina, US).

### 2.8 RNA sequencing analysis

Long RNA‐seq raw reads (dataset: Me‐X04 microglia) were processed individually or by using the nf‐core workflow^17^ (3.0 version of “rnaseq”). Reads were trimmed and filtered using Trim Galore (v.0.4.4)^18^ with a Phred quality score cutoff 30. Processed reads were aligned to the genome assembly GRCm38 using STAR version 2.5.4b^19^ with options –outFilterMismatchNoverLmax 0.04 and –outFilterMultimapNmax 10. Aligned reads mapping to features were assigned using featureCounts (Rsubread 1.32.4)^20^ using the Gencode M16 GTF. Reads were filtered using the function “filterByExpr” to remove lowly expressed genes. Individual library sizes were normalised using trimmed mean of M‐values (TMM).^21^ Principal component analysis and unsupervised consensus clustering with Cola^22^ was used to detect outliers and sample's features inducing batch effect.

Small RNA‐seq reads (dataset: Me‐X04 microglia) were trimmed, aligned, and mapped using the Qiagen Data Analysis Center (Qiagen, Hilden, Germany). Unique Molecular Identifier (UMI) corrected reads were subsequently used for differential gene expression (DGE).

Differential expression was estimated using quasi‐likelihood F‐testing with edgeR (3.24.3).^23^ Transcripts with a *log2 FC* of ≥ 0.7 or ≤ −0.7 and *FDR* < 0.05 were considered differentially expressed. Heatmaps were produced using ComplexHeatmap^24^ with hierarchal clustering performed using Ward's least absolute error with Manhattan distance.^25^

Bulk RNA sequences of mRNA (dataset: BV‐2) were aligned and quantified to the mouse genome of reference GRCm38 using the nf‐core workflow^17^ (3.0 version of “rnaseq”). Lowly expressed molecules in any condition were filtered using the function “filterByExpr” to increase the reliability of the mean‐variance relationship. Differences between samples due to the sequencing depth were minimized normalizing the count using the TMM^21^ method and applied a log transformation minimizing sum of sample‐specific squared difference to enhance the true positive and negative ratio in the downstream analysis.^26^ Principal component analysis and unsupervised consensus clustering with Cola^22^ was used to detect outliers and sample's features inducing batch effect. We finally created the design matrix for each pair of conditions to compare (contrast) and performed the differential expression analysis using limma/edgeR model^27^ controlling for the false discovery rate with Benjamini‐Hochberg Procedure.^28^

The same pipeline above was used to analyze multiple external RNA sequencing data and obtain the microglia‐specific proinflammatory list of genes. The count matrices derived from GSE109834 (sorted microglial cells with LPS stimulation), GSE123021 (homeostatic microglial cells), GSE75246 (sorted microglial cells with LPS stimulation), and GSE98401 (sorted microglial cells with INF‐1 induction) were combined. Subsequently, the comparison between proinflammatory microglial expression profiles treated with INF‐1 induction against the LPS profiles resulted in differentially expressed genes chosen to compose the proinflammatory list.

### 2.9 Venn diagram

Differentially expressed genes of the contrast (pTGvsTG) and DAM markers of Keren‐Shaul *et al*.^9^ have been compared to get the genes present in both lists. Only protein coding genes were considered, and the intersection between the two set of gene names was determined.

### 2.10 Functional enrichment analysis

We uploaded the differentially expressed genes of each contrast to QIAGEN IPA (QIAGEN Inc., https://digitalinsights.qiagen.com/IPA)^29^ and Metascape^30^ for ingenuity pathway analysis (IPA) and functional enrichment analysis, respectively. The analysis was performed with default parameters, and IPA's background was composed of non‐differentially expressed genes. Output flagged as “bias” was disregarded.

### 2.11 miRWalk analysis

We associated the significantly deregulated miRNAs and differentially expressed genes of phagocytic Me‐X04^+^ cells (Supplementary Table S2). We obtained annotated miRNA‐Target interactions with at least 99% of binding probability from 3.0 miRWalk^31^, ^32^ database. We used the annotations and linked the differentially expressed miRNAs and genes by anticorrelation (e.g., significantly deregulated miRNA with positive log‐fold change is associated to significantly deregulated genes with negative log‐fold change).

### 2.12 Empirical cumulative distribution function of microRNA effects

Using the empirical Cumulative Distribution Function (eCDF), we conducted a test to examine the assumption of anticorrelation between each differentially expressed miRNA in phagocytic Me‐X04^+^ cells and its miRWalk gene targets. To do this, we considered both the target and non‐target genes included in the differential expression analysis. We compared the eCDF (control function) of non‐target genes based on log‐fold change values with the one of the miRNA's targets, using the Kolmogorov‐Smirnov test^33^ to test their equality. We measured the magnitude and direction of the shift of the eCDF of the targets compared to the non‐targets by calculating the area between the two curves following this formula:$\int \{F_{Y}\left(t\right)-F_{X}\left(t\right)\}\;dt$ and the Wasserstein distance.^34^

### 2.13 Spatial sequencing

#### 2.13.1 Sample preparation

We performed spatial sequencing on five‐month‐old 5xFAD transgenic (*n* = 3) and wild‐type (*n* = 2) mice. Anesthetized mice were transcardially perfused with cold saline solution with heparin 2500 IU/l (Leo Pharma A/S). The right hemisphere was collected and freshly frozen for maximum 1 min in isopentane equilibrated for 10 min in liquid nitrogen. The samples were then transferred to dry ice and stored at –80C. The brains were cut to 10 μm thick sections on PDL‐coated special glass provided by Resolve Biosciences GmbH, the company performing the spatial transcriptomic assay. *N* = 3 5xFAD transgenic and *n* = 2 wild‐type tissue samples were probed with 100 genes comprising the miRWalk targets identified in this study, DAM transition markers, and proinflammatory gene list (Supplementary Table S3). The output provided coordinates for each gene at single cell resolution and DAPI nuclei staining. In addition, the brain slices were subsequently stained from Resolve Biosciences with 1 μM pFTAA (amyloid staining) and imaged.

#### 2.13.2 Spatial sequencing data analysis

The set of data produced by the sequencing was composed of a DAPI image identifying nuclei of the cells, a DAPI image with the amyloid deposits, and a matrix with the coordinates of every transcript captured in the mouse brain of the same DAPI images. This set of data was produced for each of the mouse brain in the study. Each set of data has been processed in the following way. We imported the DAPI image in QuPath^35^ as fluorescence image with pixel width equal to 0.138 μm. We then used the function “cell detection” (setting the pixel size equal to 0.25, background radius 8, sigma 1.7, minimum area 10, maximum area 400, threshold 25, and cell expansion 10) to identify each nucleus in the image. The segmented image has been then given in input to ImageJ.^36^ We flatten the image, converted in eight‐bit, made binary, selected the segmented nuclei and saved the position of the nuclei's centers in x‐y coordinates. These operations have been performed both to localize the position of the nuclei and the deposit localization within the corresponding DAPI images. We moved to assign each transcript to a nucleus based on their coordinates. We counted a transcript belonging to a nucleus if closer than 10 μm. We repeated the operation between the transcripts and the deposits. After the quantification of the transcripts per nucleus/deposit per brain region per mouse, we moved to analyze the count matrix assembled with the single‐cell expression profiles obtained from all the datasets. We filtered the cells not expressing at least 30 transcripts and the transcripts that were not expressed in at least 100 cells. We annotated the microglial cells based on the expression of the marker *Aif1*. We compared the expression of each transcript between mouse conditions (e.g., TG vs. WT), brain regions (cortex and hippocampus), and cell types (e.g., microglia vs. non‐microglia) with the Wilcoxon rank sum test. We computed each cell distance from the closest deposit and assessed changes in the expression of each transcript in different genotypes, brain regions, and cell types. We estimated the change points of the expression in relation to the distance to establish where transcripts were changing most frequently using the “segmented” R package which implements the interval estimation for the breakpoint discussed in Muggeo 2017.^37^ We finally used the result of the change point analysis to classify the cells based on their distance.

#### 2.13.3 Spatial sequencing visualization

Black and white fluorescence images with DAPI and pFTAA staining were provided by Resolve Biosciences GmbH. To achieve images presenting both stainings, the images were merged and DAPI was visualized as blue, whereas pFTAA was visualized as yellow. To visualize the individual transcripts molecules in the images, the images were opened in the Polylux V1.6.1 tool in ImageJ (1.53f51, Java 1.80_172). Transcript files acquired from Resolve Biosciences GmbH were loaded in the tool, and the files contained the x,y, and z coordinates of the transcripts along with the corresponding gene names. Distinct colors were assigned to each gene so that genes belonging to the same group were presented with different shades and tones of the same color. The miRWALK genes (*n* = 28) were visualized with tones of blue, and the DAM genes (*n* = 41) with different tones of red. For these genes, the diameter of the transcripts was set to 7 using the Polylux tool. For greater visualization of the microglia, the transcript of *Aif1* was visualized with brighter orange, and the diameter of transcripts was set to 19. Snapshots were then acquired from specific locations.

### 2.14 Deconvolution analysis with single cell RNA sequencing

The Keren‐Shaul *et al*. single‐cell RNA sequencing (scRNA‐seq)^9^ (GSE98969) dataset has been analyzed with Seurat.^38^ We removed genes which were not expressed in at least three cells and removed those cells not expressing at least two hundred genes.^39^ Any cell expressing more than five percent of mitochondrial genes was considered dying and removed from the analysis.^40^ A final filtering has been performed based on original author's threshold on the number of unique molecular identifiers mapped per cell. Next, we normalized to reduce technical differences,^41^ scaled,^39^ and clustered the cells’ expression profiles. Precisely, we performed PCA and JackStraw techniques with the 2000 most variable genes, and we chose the number of PCA components based on the “elbow” produced by the performances of the principal components. The first 20 components guaranteed the best separation of the cells and clusters have been detected with a resolution equal to 0.2. Finally, the annotation of the clusters in specific cell‐types was solved using the gene set variation analysis (GSVA).^42^ The latter has been applied as defined by J. Javier *et al.*,^43^ and the tested gene sets have been composed by Keren‐Shaul *et al.* specific most expressed genes published in the supplementary material of the original paper.^9^

After the cell clustering, we obtained the cell‐specific statistics of the genes included in our spatial sequencing. Next, we selected the DAM microglial cells and re‐performed PCA and JackStraw based on the miRWalk phagocytic mirna target genes that were both strongly expressed in the DAM signature and in the cortex microglial cells of our spatial sequencing describing 5xFAD TG mice. As result, the original DAM cluster divided into two subpopulations.

### 2.15 Fluorescent in situ hybridization and immunohistochemistry

5xFAD mice were transcardially perfused with heparinized 0.9% saline (2500 IU/ml) at the age of 6 months. Brains were fixed with 4% PFA for 22 h and then cryoprotected with 30% sucrose in 0.1 M phosphate buffer (PB), pH 7.4 for 48 h, after which they were frozen on liquid nitrogen. Frozen brains were cut sagittally to 20 μm thick sections using a cryostat (Leica Microsystems, Wetzlar, Germany). Sections were stored at −20°C in anti‐freeze (15% sucrose, 30% ethylene glycol in PB) solution. miRNA fluorescent in situ hybridization (FISH) was done using QuantiGene ViewRNA Tissue Fluorescense Assay (Invitrogen, Thermo Fisher Scientific) according to manufacturer's instruction with some modifications. All reagents were prepared using RNAse‐free DEPC‐treated water. First, anti‐freeze solution was washed out from the sections with 0.1 M PB 5 × 30 min at room temperature and 1x over night at +4°C after which the sections were lifted to microscopic slides and hydrophobic barrier was drawn around them with ImmEdge Pen (Vector). Sections were rehydrated first with PBS and then with PBS‐Tween 20 (PBST). Permeabilization was done first with heated (92°C) 0.01 M trisodium citrate, pH 6.0, after which sections were incubated for 10 min at +40°C with Protease K. Then, the sections were washed 3 × 5 min with PBST, fixed for 5 min with 4% PFA at room temperature, and washed again 3 × 5 min with PBST. Hybridization of miR‐7 probe type 4 (VM4‐10776, Affymetrix), diluted 1:40, was done overnight at +40°C. Probe diluent was used as a negative control. Next day, the sections were washed 3 × 5 min with Wash Buffer from the kit, preamplified for 30 min at +40°C, and amplified for 30 min at +40°C, after which the label probe type 4 ViewRNA Tissue Alexa Fluor 488 (QVT0688B, Thermo Scientific™) was added for 30 min at +40°C. The sections were washed 2 × 5 min and 1 × 10 min with wash buffer and then incubated for 1 h in mouse‐on‐mouse blocking reagent (MKB‐2213, Vector), 1 drop in 1.25 ml of PBST. After 3 × 5 min washes in PBST, sections were blocked with 10% normal goat serum (NGS) in PBST for 1 h, after which primary antibodies (anti‐Iba‐1, made in rabbit, 1:300, Wako 019‐19741; anti‐amyloid‐β, clone WO‐2, made in mouse, 1:1000, Millipore MABN10) were added for overnight incubation at room temperature. The next day, the sections were washed again 3 × 5 min with PBST and secondary antibodies were added 1:500 each (Alexa Fluor 568 goat anti‐rabbit, Life Technologies A11011, and Alexa Fluor 647 donkey anti‐mouse, Life Technologies A32787) for 2 h incubation at room temperature. After 3 × 5 min washes with PBST, Vectashield mounting medium with DAPI (H‐1000, Vector) was added and the sections were covered with coverslips.

#### 2.15.1 FISH visualization and analysis

Cortices were imaged by using Leica Thunder Imager 3D Tissue Slide scanner with 40x objective. Measurement of the microglia and Aß deposits were done with ImageJ 1.53f5 (Java 1.8.0_172). The brightness of the images was first increased to have clear vision of the colocalization (white color) of the Iba‐1 staining (red) and miR‐7 staining (green). The distance from the core of the WO‐2 immunoreactive Aβ deposit to the miR‐7‐immunoreactive microglia was first drawn using the line tool in ImageJ and then the length of the line was measured to achieve the distance in μm. For the analysis purposes, distances of 100 microglia were measured from each section. Distances were measured from four transgenic animals with two sections from each. Thereby, distances of 771 microglia were measured in the end. In addition, representative images were acquired by using ZEISS LSM800 Airyscan. Images were taken with z‐stack with 1.00 μm interval and stacked together using maximum intensity projection. This part of the work was carried out with the support of UEF Cell and Tissue Imaging Unit, University of Eastern Finland, Biocenter Kuopio and Biocenter Finland.

### 2.16 *Post mortem* human brain tissue

*Post mortem* human brain tissues were acquired from the Netherlands Brain Bank (NBB), Netherlands Institute for Neuroscience, Amsterdam. All tissue and clinical information were obtained with written informed consent for research purposes, specifically brain autopsy and usage of brain tissue. Relevant details, including age, gender, *post mortem* delay (PMD), Braak stage, brain region, and diagnosis for all cases included in this investigation are available in Supplementary Table S4.

### 2.17 Statistical analysis

Graphs and statistical analysis were performed in GraphPad Prism 9. In the figure legend, the parameters of replicates (*n*), statistical test, and *p*‐value obtained are specified. Where not specified, *p*‐value was not statistically significant (*p* > 0.05). We refer to *n* in the animal samples as single biological replicate (mouse) and in BV‐2 cell line experiment as replicates of different independent experiments.

## 3 RESULTS

### 3.1 Result 1

#### 3.1.1 Phagocytic microglial activation primes changes in gene expression in Alzheimer's mice

**FIGURE 1:**
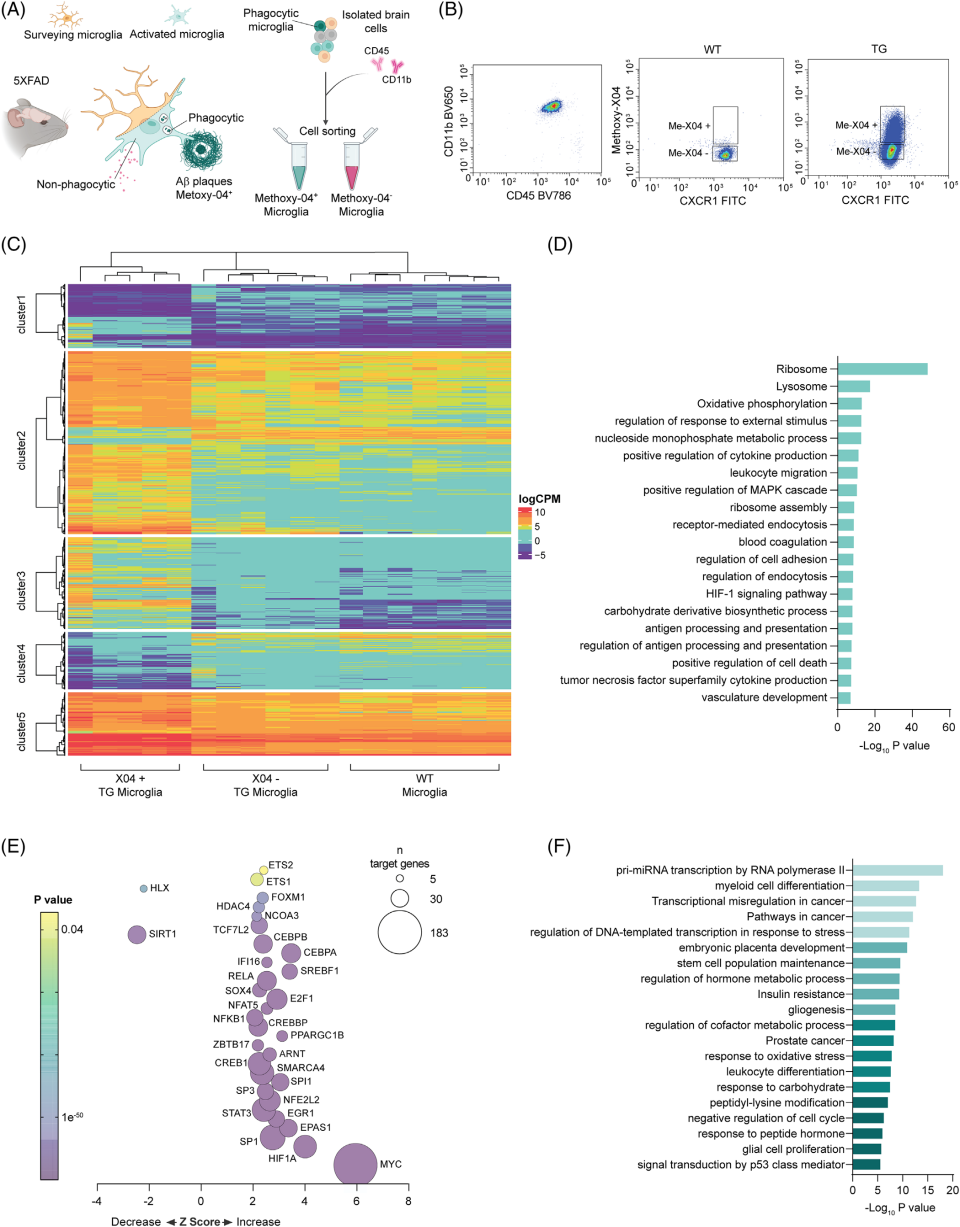
Phagocytic microglial activation primes changes in gene expression in Alzheimer's mice. (A) Schematic representation of the experimental design. Six‐month old Alzheimer's disease (AD) 5xFAD mice were injected with permeable amyloid deposit staining fluorescent probe Methoxy‐X04 (Me‐X04) before microglia fluorescent activated cell sorting (FACS) cell sorting with Cd11b and Cd45 markers. Phagocytic microglia (Me‐X04 positive) were separated by non‐phagocytic (Me‐X04 negative) and sequenced to detect differential expression of transcripts. (B) Representative plot of FACS sorting of 10.000 events of the microglia double positive population (Cd11b, Cd45) divided for Me‐X04 positivity. (C) Heat map of logCPM of detected transcripts between murine Alzheimer's phagocytic microglia ( TG Me‐X04^+^), Alzheimer's non‐phagocytic (TG Me‐X04^−^) and wild‐type (WT) (*n* = 6 TG and 7 WT animals as biological replicates, hierarchal clustering performed using Ward's least absolute error with Manhattan distance). (D) Bar plot of functional enrichment analysis top 20 significant Metascape clusters performed on differentially expressed transcripts in phagocytic versus non‐phagocytic AD microglia. (E) Bubble plot of Ingenuity pathway analysis (IPA) of upstream regulators (transcription factors) based on differentially expressed genes between phagocytic versus non‐phagocytic AD microglia obtained through RNA‐seq. *Z‐*score indicates IPA prediction of pathway activation (positive value) or inhibition (negative value) in phagocytic microglia. Color bar expresses *p*‐value significancy (yellow to purple), size expresses the number of genes contained in the pathway (|*z* | > 2 is considered significant, pathway containing > 10 genes were included). (F) Bar plot of functional enrichment analysis top 20 significant Metascape clusters performed on differentially expressed transcription factors in phagocytic AD versus non‐phagocytic microglia.

To determine the transcriptional signature of phagocytic microglia in the brain of the 5xFAD mice, 5XFAD transgenic mice (TG) and their wild‐type (WT) controls were injected with the brain permeable Methoxy‐X04 (Me‐X04) dye^44^ to stain the protein ß‐sheet structures of Aβ aggregates prior to the isolation of microglia through fluorescent activated cell sorting (FACS) based on their uptake of the Me‐X04 dye (Figure 1A). As previously described,^10^ microglia were identified as Cd11b^+^ and Cd45^+^ population which were devoid of Me‐X04 due to the absence of Aβ aggregates in wild‐type animals. In contrast, transgenic 5xFAD (AD) Cd11b^+^ and Cd45^+^ microglial population was sorted into Me‐X04^−^ (non‐phagocytic) and Me‐X04^+^ (phagocytic) microglia (Figure 1B, Supplementary Figure S2).

Bulk RNA‐sequencing of total RNA of the sorted TG phagocytic and non‐phagocytic microglia from 5XFAD mice revealed 1206 differentially expressed (DE) genes (Supplementary Table S5). Surprisingly, only 45 genes were found to be altered between WT and TG non‐phagocytic microglia, suggesting that changes in gene expression are dictated mostly by intake of Aβ rather than the AD genotype itself (Figure 1C, Supplementary Table S5). Among the 45 DE genes found in the WT versus TG non‐phagocytic microglia, 42 exhibited similar differential expression patterns in TG phagocytic and non‐phagocytic microglia (upregulated or downregulated) (Supplementary Table S5). Notably, some of these genes, such as *ApoE, Axl, Clec7a, Csf1, Cst7, Egr1, Itgax, Lpl*, and *Lyz2* are well established DAM marker genes.^9^ Hierarchical cluster analysis of these DE transcripts in TG phagocytic microglia (Figure 1C) highlighted distinct gene expression patterns: genes in the gene sets 1 and 4 showed low expression level in WT and TG non‐phagocytic microglia, with even further downregulation in phagocytic microglia. Conversely, genes in the gene sets 2 and 5 displayed high expression in WT and TG non‐phagocytic microglia, which were even higher in the TG phagocytic microglia. Gene set 3 consisted of genes exclusive to TG phagocytic microglia, showing highly anticorrelated expression with respect to WT and TG non‐phagocytic microglia. Interestingly, functional enrichment analysis of the genes in the gene set 3 revealed their involvement in lysosomal functions and a strong association with AD (*p* < 0.05), suggesting that this gene subset might be crucial for microglial phagocytosis in AD (Supplementary Figure S3).

Functional enrichment analysis of the 1206 genes (784 upregulated and 422 downregulated) in AD phagocytic microglia confirmed their association with phagocytic activity (*Lysosome*) and RNA translation (Figure 1D), consistent with previously published data^9^, ^10^. These findings together validated the successful isolation of phagocytic microglia and that gene expression changes in microglia are primarily driven by functional changes rather than AD genotype.

In 2017, Keren‐Shaul *et al.* defined the DAM signature in microglia that identifies a subtype of microglia which may restrict AD‐related neurodegeneration.^9^ Comparing the gene expression profile in phagocytic Me‐X04^+^ microglia to the published DAM signature revealed an overlap of 260 genes out of the total 345 DAM genes (Supplementary Figure S4A), suggesting that more than 70% percent of the DAM genes are also differentially expressed in the phagocytic Me‐X04^+^ microglia. The shared set of genes (260) were functionally enriched in previously identified key function of AD‐induced phagocytic microglia, including translation and lysosomal activity, as well as HIF‐1α pathway, associated with synapse loss in AD^13^ (Supplementary Figure S4B).

We next analyzed whether the activation of specific transcription factors may underlie the differences in mRNA expression between phagocytic and non‐phagocytic AD microglia. IPA of upstream regulators revealed the activation of 30 and inactivation of 2 transcription factors specifically in the Me‐X04^+^ microglia in the 5xFAD mouse brain (Figure 1E, Supplementary Table S6). Notably, GATA3 was the only transcription factor uniquely activated between TG Me‐X04^−^ and WT microglia (Supplementary Table S6). Functional enrichment analysis of transcription factors in Me‐X04^+^ microglia revealed the involvement of several transcription factors, including CEBPA, EGR1, ETS1, HDAC4, HIF1A, MYC, NFKB1, RELA, SMARCA4, SP3, SPI1, SREBF1, STAT3, and TCF7L2 in the transcriptional regulation of miRNA precursors (Figure 1F), a powerful class of non‐coding RNAs that regulate sudden changes in gene expression post‐transcriptionally.

### 3.2 Result 2

#### 3.2.1 miRNAs are involved in the Alzheimer's‐induced microglia phagocytic phenotype

**FIGURE 2:**
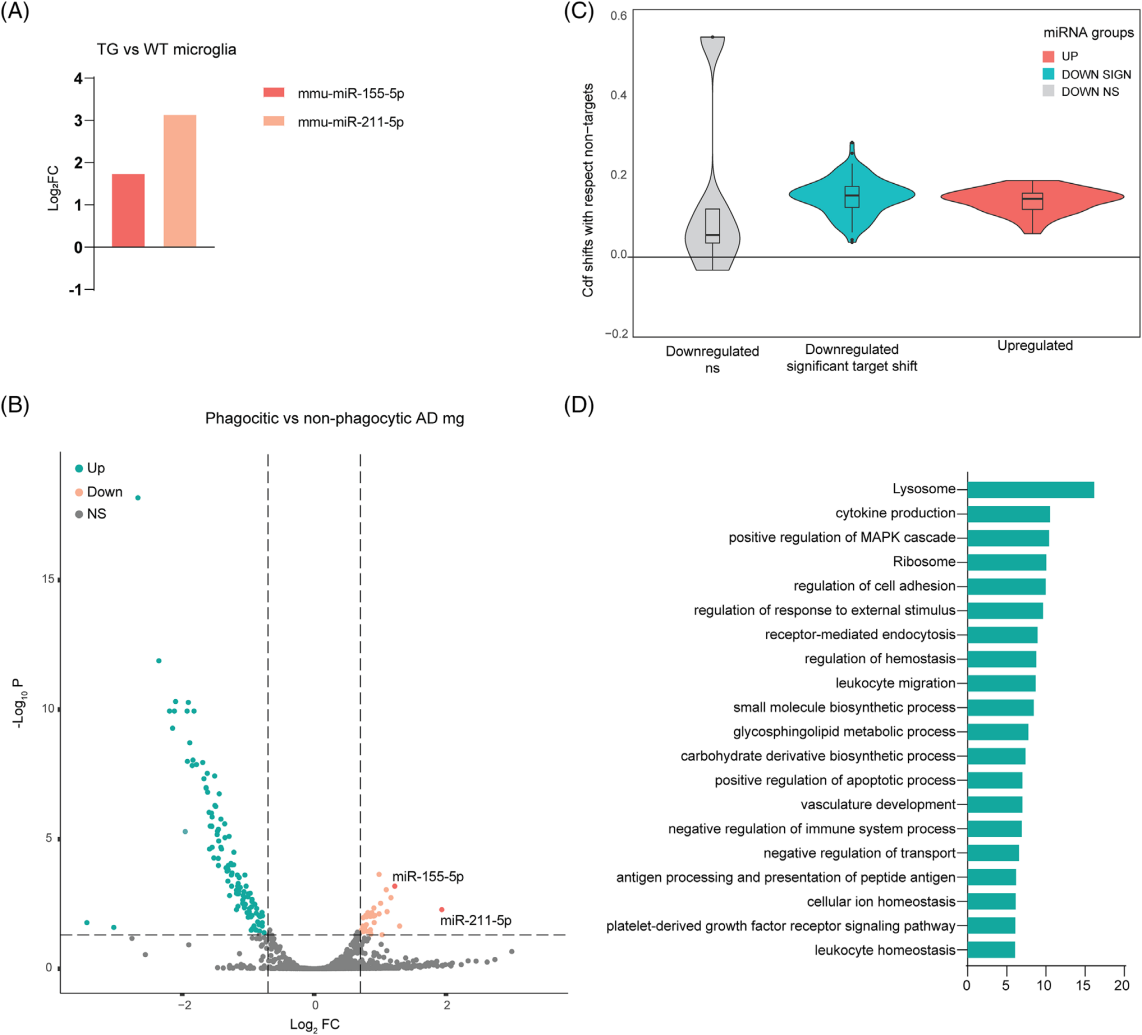
MicroRNAs (miRNAs) are involved in the Alzheimer's induced microglia phagocytic phenotype. (A) Bar plot of *log2FC* of the only two differentially expressed miRNAs in Alzheimer's disease (AD) non‐phagocytic microglia versus wild‐type (WT) microglia *mmu‐miR‐155‐5p* (dark orange) and *mmu‐miR‐211‐5p* (light orange) obtained from RNA‐seq of 5xFAD fluorescent activated cell sorting (FACS) sorted microglia (*n* = 6 transgenic [TG] and 7 WT animals as biological replicates; *FDR* < 0.01 and |*log2FC* | > 1). (B) Volcano plot of differentially expressed miRNAs in AD phagocytic versus non‐phagocytic microglia. Highlighted significantly upregulated (light orange) and downregulated (green) miRNAs, miRNAs upregulated in non‐phagocytic AD microglia versus WT (dark orange) (*n* = 6 TG animals as biological replicates; *FDR* < 0.01 and |*log2FC* | > 1). (C) Violin plot of the shifts in expression of miRNA targets where each one measures how much the cumulative distribution function of an miRNA's differentially expressed targets separates from the function of the non‐targets. The shifts have been categorized based on the type of miRNA regulation (non/significant and up/downregulated). Each miRNA CDF curve is obtained with the single specific miRNA targets against all the non‐targets (*n* = 6 TG animals as biological replicates; *p*‐value calculated with Kolmogorov‐Smirnov test). (D) Bar plot of functional enrichment analysis top 20 significant Metascape clusters performed on differentially expressed miRWalk targets of the significantly differentially expressed miRNAs in AD phagocytic versus non‐phagocytic microglia.

As we observed functional connections between transcription factors and miRNA transcription in phagocytic microglia, we conducted small RNAs sequencing on brain tissue of WT and TG mice, isolating FACS‐sorted non‐phagocytic and phagocytic (Me‐X04^−/+^ respectively) microglia. The analysis of this dataset revealed only the upregulation of two miRNAs in TG Me‐X04^−^ microglia compared to WT Me‐X04^−^ microglia: *miR‐155‐5p* and *miR‐211‐5p* (Figure 2A, Supplementary Table S7). Both *miR‐155‐5p* and *miR‐211‐5p* were also upregulated in TG Me‐X04^+^ phagocytic microglia.

In contrast, phagocytic Me‐X04^+^ microglia exhibited 151 DE miRNAs, with 118 being down‐regulated and only 33 up‐regulated compared to non‐phagocytic microglia (Figure 2B, Supplementary Table S7). A total of 77% of the DE miRNAs in Me‐X04^+^ microglia were downregulated, implying a consequential derepression of their mRNA targets. Through miRWalk algorithm analysis,^31^, ^32^ we obtained validated and predicted targets for the Me‐X04^+^ microglia DE miRNAs, filtering for binding score higher than 0.99. Cumulative distribution analysis of the miRNA‐target pairs, divided into downregulated and upregulated miRNAs, revealed that downregulated miRNAs present a subclass that significantly derepressed their mRNA targets. On the other hand, the small set of upregulated miRNAs did not show a canonical repressive function (Figure 2C, Supplementary Table S2). Further analysis involved the selection of mRNAs that showed anticorrelation with the miRNA expression. Functional enrichment analysis of all the anticorrelated DE miRNA targets in the Me‐X04^+^ microglia indicated an enrichment in GO‐terms related to *Ribosomal* and *Lysosomal* *function*s, further highlighting their potential role in phagocytic and gene regulatory activities. Among the 151 DE miRNAs, 110 targeted both predicted and validated mRNAs involved in phagocytosis, implying that more than 70% of DE miRNAs were indeed involved in regulating the phagocytic phenotype through both canonical and non‐canonical mechanisms (Figure 2D). Given our focus on post‐transcriptional regulators of phagocytic microglia, we selected anticorrelated miRNA‐mRNAs pairs based on the enrichment of the mRNA targets in the *Lysosome* pathway for more detailed analysis.

### 3.3 Result 3

#### 3.3.1 miRNAs affect phagocytosis and microglia fitness in vitro

We selected the most prominently differentially expressed miRNAs in phagocytic microglia for further investigation for their phagocytosis‐regulating properties in vitro. To ensure a relevant cellular system for functional assays, we took advantage of previously generated small RNA sequencing data^45^ (GSE123262) to exclude any unexpressed miRNAs in our reference cellular system (BV‐2 microglial cell line). Additionally, to increase the translational value of our research, we focused on miRNAs conserved between mouse and human. Following these criteria, we established a list of 9 miRNAs (*miR‐7a‐5p, miR‐29a‐3p, miR‐146a‐5p, let‐7g‐5p, miR‐140‐5p, miR‐148b‐3p, miR‐339‐5p, miR‐532‐3p, miR‐744‐5p*), all of which were downregulated in our phagocytic microglia dataset and had a significant derepressive effect on mRNA targets (Supplementary Figure S5).

**FIGURE 3:**
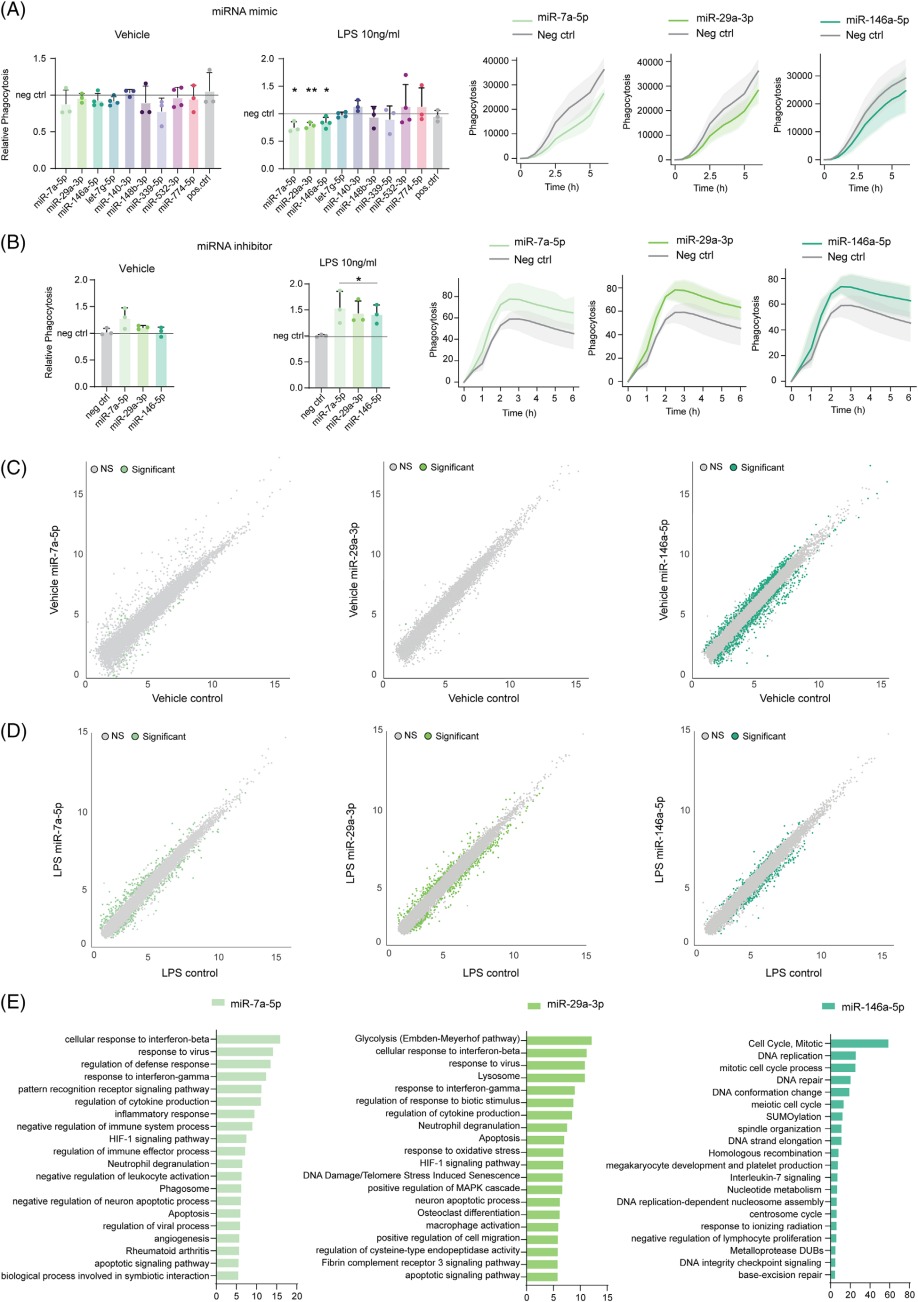
MicroRNAs (miRNAs) affect phagocytosis and microglia fitness in vitro. (A) *(left)* Live imaging phagocytic assay at 6 h timepoint of BV‐2 cells overexpressing single miRNAs in respect of scramble control (gray line) treated with lipopolysaccharide (LPS) 10 ng/ml for 24 h or vehicle media. (*right*) Time point data of live imaging phagocytic assay of BV‐2 cells treated with LPS 10 ng/ml for 24 h and overexpressing single miRNAs (green) in respect of scramble control (gray). Data are calculated as a relative fluorescence after background subtraction and normalized on confluency and shown as mean ± SD (*n* = 3 or 4 independent experiments as shown by the single data point; * = *p*‐value < 0.05, ** = *p*‐value < 0.01 calculated with two tailed Student t‐test). (B) *(left)* Live imaging phagocytic assay at 6 h timepoint of BV‐2 cells treated with miRNA inhibitors in respect of scramble inhibitor (gray line) treated with LPS 10 ng/ml for 24 h or vehicle media. (*right*) Time point data of live imaging phagocytic assay of BV‐2 cells treated with LPS 10 ng/ml for 24 h and miRNA inhibitors of single miRNAs (green) in respect of scramble inhibitor (gray). Data are calculated as a relative fluorescence after background subtraction and normalized on confluency and shown as mean ± SD (*n* = 3 or 4 independent experiments as shown by the single data point; * = *p*‐value < 0.05, ** = *p*‐value < 0.01 calculated with two tailed Student t‐test). (C) Scatter plot of differentially expressed genes in BV‐2 cells overexpressing *miR‐7a‐5p* (light green), *miR‐29a‐3p* (medium green), *miR‐146a‐5p* (dark green) in respect of scramble negative control (gray) transfected cells (*n* = 4 independent experiments; adj.*p*‐value < 0.05 and|*log2FC* | > 0.3). (D) Scatter plot of differentially expressed genes in BV‐2 cells treated with LPS for 24 h and overexpressing *miR‐7a‐5p* (light green), *miR‐29a‐3p* (medium green), *miR‐146a‐5p* (dark green) in respect of LPS treated scramble negative control transfected cells (*n* = 4 independent experiments; adj.*p*‐value < 0.05 and|*log2FC* | > 0.3). (E) Bar plot of functional enrichment analysis top 20 significant Metascape clusters performed on differentially expressed genes in LPS treated BV‐2 cells for 24 h overexpressing *miR‐7a‐5p* (light green), *miR‐29a‐3p* (medium green), *miR‐146a‐5p* (dark green) in respect of LPS treated scramble negative control transfected cells (*n* = 4 independent experiments).

To test whether these miRNAs affect phagocytosis in BV‐2 cells, we transfected this cell line with miRNA mimics and performed live fluorescent bead phagocytosis assay. None of the miRNAs were able to affect the basal level of phagocytosis compared to the scrambled control (Figure 3A). As the 5xFAD brain microglia miRNA and mRNAs data show that changes in gene expression strongly depends on microglial activation state, we stimulated BV‐2 cells in vitro with lipopolysaccharide (LPS). In these conditions, we detected a significant decrease in phagocytosis upon overexpression of *miR‐7a‐5p*, *miR‐29a‐3p*, *miR‐146a‐5p* (Figure 3B). These results aligned with the Me‐X04^+^ microglia RNA‐seq data, where these miRNAs where downregulated, suggesting their involvement in regulating microglia phagocytosis.

To further confirm the specific role of these miRNAs in regulating microglia phagocytosis, we performed live fluorescent bead phagocytosis assay on BV‐2 cells transfected with inhibitors to *miR‐7a‐5p*, *miR‐29a‐3p*, *miR‐146a‐5p*. Consistent with our earlier observations, none of the miRNA inhibitors showed any significant effect on the basal level of phagocytosis when compared to the control inhibitor (scrambled) (Figure 3B). However, all three miRNA inhibitors exhibited an enhanced phagocytic response in BV‐2 cells treated with LPS.

To elucidate the functionality of these three miRNAs based on their targets, we performed mRNA‐seq of BV‐2 cells overexpressing *miR‐7a‐5p*, *miR‐29a‐3p* or *miR‐146a‐5p* with or without LPS stimulation (Supplementary Table S8). In accordance with our earlier findings, the overexpression of *miR‐7a‐5p*, *miR‐29a‐3p* miRNA mimics did not affect gene expression of naïve BV‐2 cells (Figure 3C), whereas LPS treatment caused significant alterations in gene expression (Figure 3D, Supplementary Table S8). Functional enrichment analysis of the differentially expressed genes in miRNA mimic transfected and LPS stimulated BV‐2 cells revealed an impact of *miR‐7a‐5p* and *miR‐29a‐3p* on genes involved in phagocytosis (GO terms *Phagosome and Lysosome*) and overall inflammatory response (Figure 3E). On the other hand, *miR‐146a‐5p* exhibited broader targeting in naïve conditions without direct effects on regulating phagocytosis (Figure 3A,E). In LPS‐stimulated cells, *miR‐146a‐5p* regulated genes involved in active cell division, proliferation, and Interleukin‐7 signaling (Figure 3E), suggesting an indirect role in phagocytosis by regulating microglial fitness and inflammatory phenotype in a context dependent manner.

These findings shed light on the functional roles of these miRNAs in regulating microglia phagocytosis and their potential implications in neuroinflammation and neurodegenerative diseases.

### 3.4 Result 4

#### 3.4.1 miRNA phagocytic target gene expression is downregulated in Iba1‐positive cells

**FIGURE 4:**
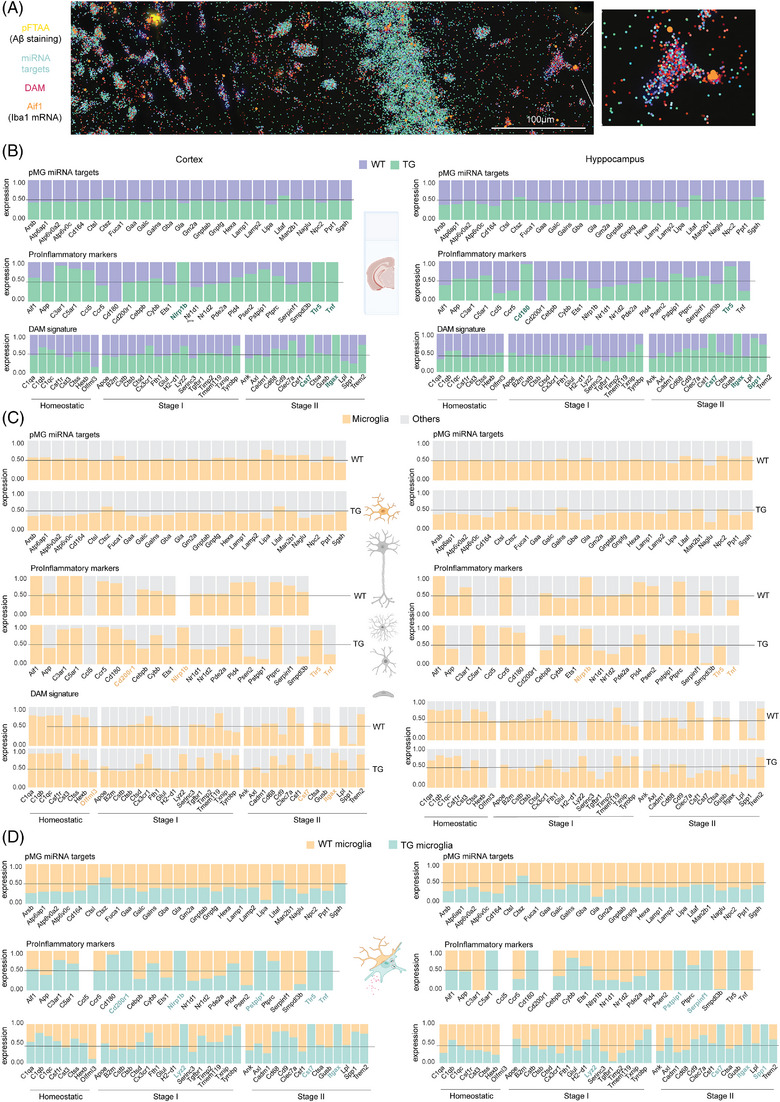
Spatial analysis of microRNA (miRNA) phagocytic targets and disease‐associated microglia (DAM) and pro‐inflammatory signature. (A) Representative image of spatial sequencing of 5xFAD brain slices. In yellow, the staining of Aβ aggregates with pFTAA; shades of red represent the selected list of DAM genes; and in shades of blue, the miRWalk targets involved in phagocytosis. In orange, *Aif1* (Iba1 coding gene) expression identifying microglia cells representatively outlined in the zoom. (B) Stacked bar plot with the average expression of miRWalk, DAM, and pro‐inflammatory genes (Supplementary Table S2) overall in 5XFAD Alzheimer's disease (AD) slices (green) with respect to wild‐type (WT) animals (purple) in cortex (*left*) and hippocampi (*right*) obtained from the spatial sequencing analysis. In green, the genes markers discussed (*n* = 2 WT and 3 transgenic [TG] animals as biological replicates). (C) Stacked bar plot with the average expression of miRWalk, DAM and pro‐inflammatory gene lists (Supplementary Table S2) in microglial cells (yellow, *Aif1* positive cells) in respect of all the other cell types (gray, *Aif1* negative) in cortex (*left*) and hippocampi (*right*) obtained from the spatial sequencing analysis. In yellow, the genes markers discussed (*n* = 2 WT and 3 TG animals as biological replicates). (D) Stacked bar plot with the average expression of miRWalk, DAM, and pro‐inflammatory gene lists (Supplementary table S2) in microglial *Aif1* positive Alzheimer's disease cells (blue) in respect of WT microglia (yellow) in cortex (*left*) and hippocampi (*right*) obtained from the spatial sequencing analysis. In blue, the genes markers discussed (*n* = 2 WT and 3 TG animals as biological replicates).

To spatially characterize microglial expression of miRNA targets involved in phagocytosis, we performed spatial transcriptomics on the brains of 5xFAD TG mice and their WT counterparts, focusing on 100 genes (Figure 4A). In addition to the phagocytic targets of *miR‐7a‐5p*, *miR‐29a‐3p*, and *miR‐146a‐5p* (*n* = 28 genes), to gain insight whether the phagocytic microglia belong to the previously described DAM subtype, we included selected DAM signature genes (*n* = 41 genes) that are involved in the transition of homeostatic microglia to DAM 1 (*Trem2* independent) and DAM 2 (*Trem2* dependent) stages^9^ (Supplementary Table S3). Moreover, to understand whether the phagocytic microglia show proinflammatory gene expression, we included a list of microglia specific pro‐inflammatory genes (*n* = 30 genes) generated by integration of RNA‐seq datasets of sorted murine microglia treated with LPS or INFγ^46^, ^47^, ^48^ (GSE109834, GSE98401, GSE75246) (Supplementary Table S3).

The analysis of the expression of phagocytic miRNA targets in the entire brain slices of TG and WT mice revealed no overall significant changes between the AD and WT mice in the cortex or hippocampi (Figure 4B, Supplementary Table S9). However, DAM and pro‐inflammatory signature exhibited a detectable enrichment of *Nlrp1b*, *Cd180, and Tnf* in 5xFAD mice (Supplementary Table S9). To analyze the expression of the miRNA target genes within microglia, we used *Aif1* gene (Iba1)^49^ as marker to identify microglia in our spatial transcriptome dataset. As expected, the DAM genes were associated with the AD genotype, with *Itgax* and *Cst7* transcripts showing strong upregulation both in cortex and hippocampi in the brains of the 5xFAD mice and being undetected in WT animals (Figure 4C,D, Supplementary Table S10, S10).

We further found that 23 out of the 26 phagocytic miRNA targets were consistently downregulated in Iba1‐positive cells in the brain of 5xFAD mice (Supplementary Table S10). Additionally, we identified five pro‐inflammatory markers (*Cd200r1, Nlrp1b, Pstpip1, Tlr5, Tnf*) of which two were specific for cortical (*Cd200r1, Tnf*) and one (*Serpinf1*) for hippocampal microglia in the 5xFAD brain. Four DAM markers were characteristic of TG microglia (*Cst7, Itgax, Lyz2)* in both regions, and one specific for hippocampal TG microglia (*Spp1)* (Supplementary Table S10).

### 3.5 Result 5

#### 3.5.1 The expression of phagocytic miRNA targets is dynamically induced in microglia in close proximity to the amyloid deposits

**FIGURE 5:**
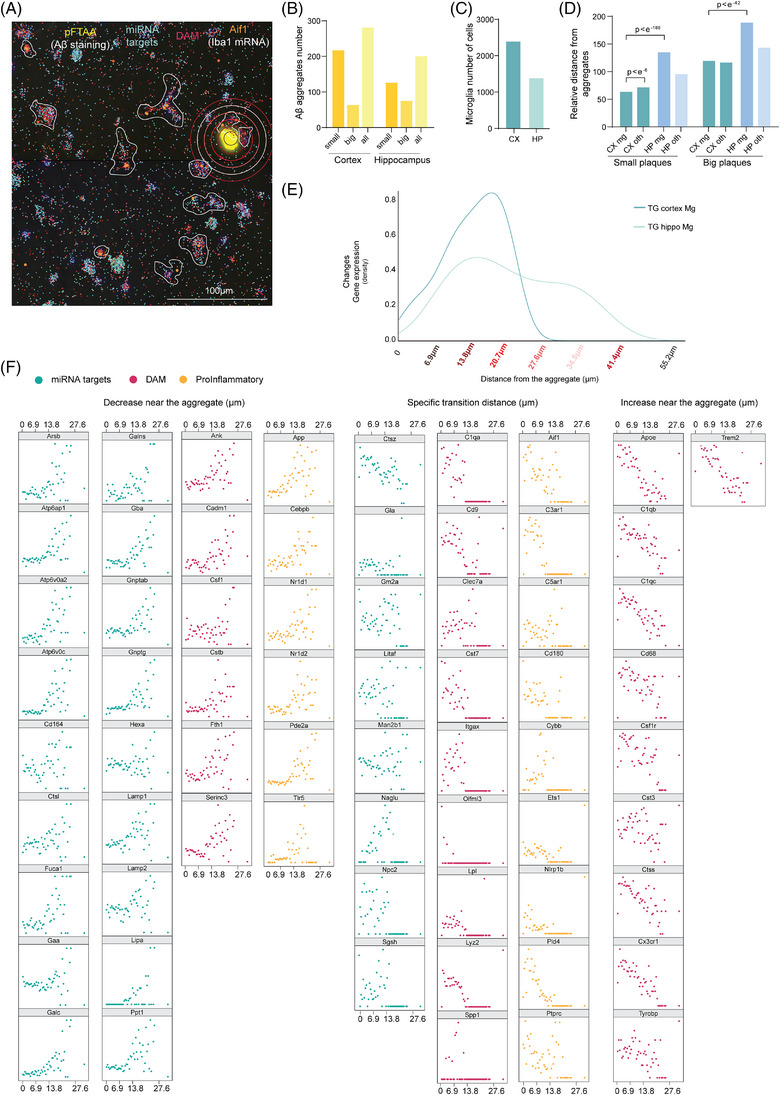
The expression of phagocytic microRNA (miRNA) targets is dynamically induced in microglia near the beta‐amyloid deposits. (A) Representative image of spatial sequencing of 5xFAD brain slices. In yellow, the staining of Aβ deposits with pFTAA; shades of red represent the selected list of disease‐associated microglia (DAM) genes; and in shades of blue, the miRWalk targets involved in phagocytosis. In orange, *Aif1* (Iba1 coding gene) expression identifying microglia cells. Circles identify the distance of the Iba1 positive cells from the deposit utilized in the analysis. (B) Bar plot of the quantification of pFTAA positive Aβ deposits in cortex and hippocampi divided into small (5–40 μm in diameter) and big (> 40 μm in diameter) (*n* = 3). (C) Bar plot of the quantification of *Aif1* positive cells (microglia) in cortex and hippocampi (*n* = 3). (D) Bar plot of the quantification of *Aif1* positive (mg) or not (oth) distance from pFTAA positive Aβ deposits in cortex and hippocampi divided into small (5 to 40 μm in diameter) and big (>40 μm in diameter) (*n* = 3 transgenic [TG] animals as biological replicates; data showed as average and *p*‐value calculated with Wilcoxon rank sum test). (E) Distribution of gene expression changes, expressed as density, in respect of the distance from the deposit in μm in cortex and hippocampi. The higher the peak, the greater degree of gene expression changes occur at the specific distance. (F) Scatter plot of the expression of the specific gene belonging to miRWalk (blue), DAM (magenta) or pro‐inflammatory (yellow) gene lists in respect of the distance of the *Aif1* positive cell (microglia) (*n* = 3 TG animals as biological replicates; data shown as normalized value between 0 and 1).

Subsequently, we stained the Aβ deposits in 5xFAD TG mice with pentameric formyl thiophene acetic acid (pFTAA),^50^ which detects protein misfolding and is used as a generic dye for Aβ deposits in these mice, and analyzed the distance of the selected microglial transcripts to the Aβ deposits (Figure 5A). As anticipated from previous literature,^51^ we observed a significantly higher number of deposits in the cortex of the 5xFAD TG mice compared to the hippocampal area, primarily due to an increase in the number of small deposits (Figure 5B). Correspondingly, we also detected a higher number of microglial cells in the cortex compared to the hippocampi^52^ (Figure 5C). Strikingly, hippocampal microglia were found to be located further away from the deposits compared to cortical microglia and any other cell type (Figure 5D), emphasizing a regional disparity in Aβ deposits accumulation and distinct microglial responses in different brain under AD‐like pathology.

We next evaluated whether the distance from the Aβ deposits influenced the expression of phagocytic miRNA target genes, DAM markers and proinflammatory gene profiles in microglia. In hippocampal microglia, the distance from the deposits did not influence the expression of these transcripts (Figure 5E, Supplementary table S11). In contrast, the expression of the transcripts in cortical microglial was affected by their distance to the deposits (Figure 5F, Supplementary table S11).

Specifically, 18 out of the 26 phagocytic miRNA targets exhibited a homogenous increase in the expression at a distance of approximately 20 μm from the Aβ deposit, followed by a sharp decrease in expression in microglia located in close proximity to the deposit. We observed the same behavior in six DAM genes (*Ank, Cadm1, Csf1, Cstb, Fth1, Serinc3*) and six pro‐inflammatory genes (*App, Cebpb, Nr1d1, Nr1d2, Pde2a, Tlr6*). No strong correlation with the distance was evident for the other eight phagocytic miRNA targets. Surprisingly, most of the DAM markers, including *ApoE, C1qb, C1qc, Cd88, Csf1r, Cst3, Ctss, Cs3cr1, Tyrobp and Trem*2 showed a similar increase in expression in close proximity to the deposits.

### 3.6 Result 6

#### 3.6.1 miRNAs phagocytic targets identify a switch in microglial states induced by close proximity to the amyloid aggregates

**FIGURE 6:**
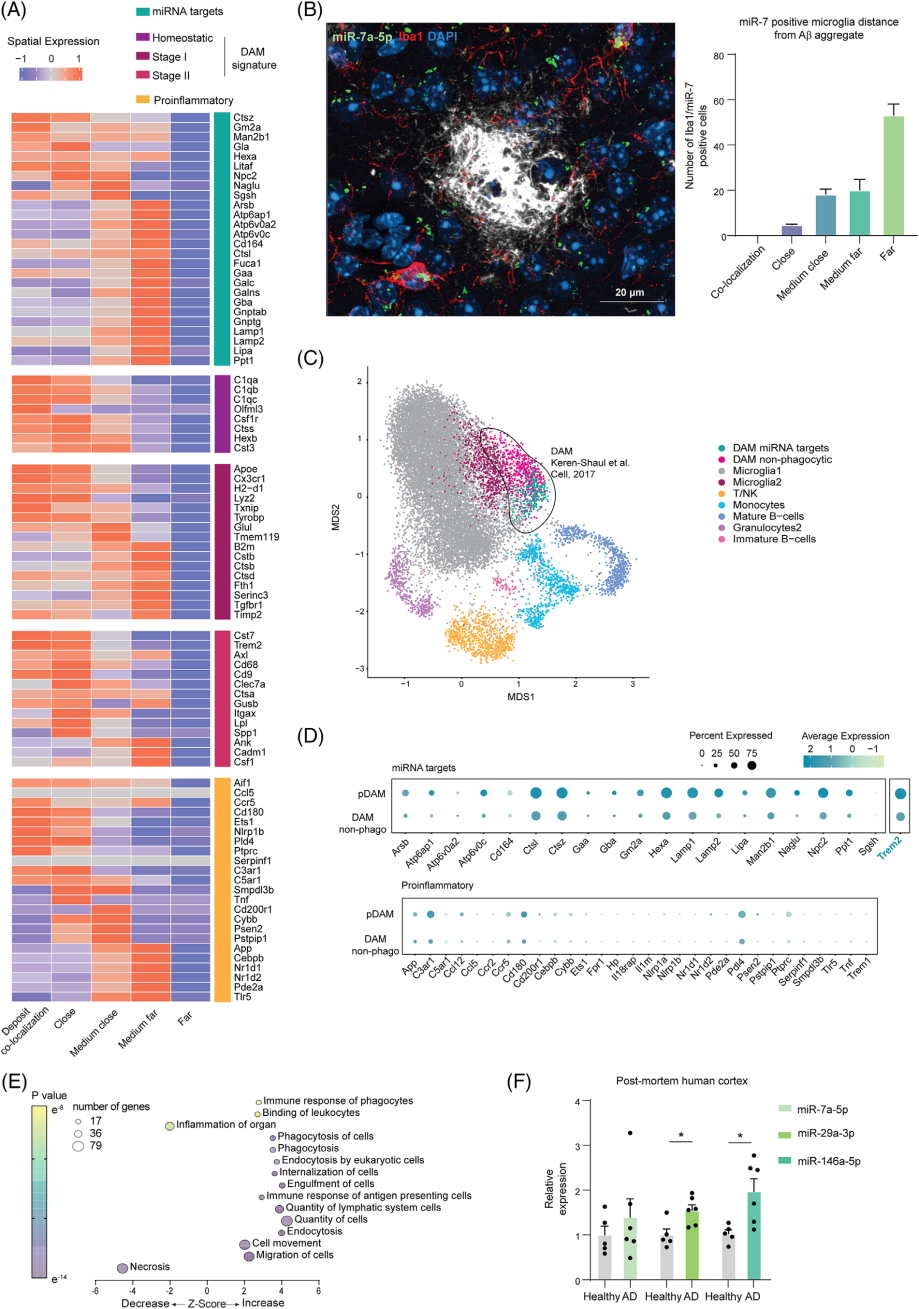
MicroRNAs (miRNAs) phagocytic targets identify a switch in microglial states in proximity of Aβ aggregates and segregate disease‐associated microglia (DAM) population. (A) Heat map of the row‐scaled average expression of the gene in a distance‐specific set of cells obtained through spatial sequencing. Gene lists were divided into miRWalk, DAM, and pro‐inflammatory (Supplementary Table S2) in microglial cells (*Aif1* positive) categorized on the approximate distance from the Aβ aggregate: co‐localization (0 μm), close (0 to 9 μm), medium‐close (9 to 17 μm), medium‐far (17 to 27 μm), far (more than 27 μm) (*n* = 3 transgenic [TG] animals as biological replicates). (B) *(left)* Representative confocal image acquired with 40x objective of fluorescence in situ hybridization and immunohistochemistry of miR‐7 (*green*), Iba1 microglia marker (*red*), amyloid‐β (*white*), and DAPI (*blue*). Merge of miR‐7 and Iba1 (yellow) identify miR‐7‐positive microglia. *(right)* Quantification of 771 total miR‐7‐positive microglia distance from Aβ aggregates obtained from four transgenic animals with two sections from each animal. Data expressed as relative percentage of double positive miR‐7 and Iba1 microglia identified at specific distance from the aggregate (n = 4 biological replicate, n = 2 slice per biological replicate; data as mean ± standard deviation). (C) U‐map obtained by Keren‐Shaul *et al.* dataset (GSE98969) highlighting in green the re‐clustering of DAM subpopulation (circled in black) expressing miRWalk target genes (DAM miRWalk targets), and in magenta DAM not expressing it. In gray, homeostatic microglia (Microglia 1), purple Stage 1 DAM (Microglia2), other immune cell clusters as T‐cells and natural killer (yellow, T/NK), monocytes (cyan), mature B cells (darker blue), granulocytes (light purple), immature B‐cells (light pink). (D) Dot plot of the average expression of miRWalk targets in the identified DAM phagocytic subpopulation (pMG) and in the non‐phagocytic DAM sub‐clustering. Below, the expression of pro‐inflammatory genes in the same two DAM subpopulations. (E) Bubble plot of ingenuity pathway analysis of genes specific of the DAM pMG population based on differentially expressed genes in respect the other microglia clusters without the original DAM markers. *Z‐*score indicates IPA prediction of pathway activation (positive value) or inhibition (negative value) in phagocytic microglia. Color bar expresses *p*‐value significancy (yellow to purple), size expresses the number of genes contained in the pathway (|*z* | > 2 is considered significant, pathway containing > 10 genes were included). (F) Bar plot of microRNA expression measured by qPCR from *post mortem* human brain tissues (Supplementary Table S4). Data shown as relative expression of Alzheimer's disease (AD) patient in respect of non‐demented control (healthy) and shown as mean ± SD (*n* = 5 healthy and *n* = 6 AD patients as shown by the single data point; * = *p*‐value < 0.05 calculated with two‐tailed Student t‐test).

We then delved deeper into the gene expression patterns of microglia at specific distances from the Aβ deposits (Figure 5F). Notably, the targets of the phagocytic Me‐X04^+^ microglia miRNAs exhibited a two stage activation at different distances from the deposits (co‐localization (0 μm) and medium far (17 to 27 μm)). Microglia closer to the deposits showed upregulation of eight of the phagocytic mirna targets (C*tsz, Gm2a, Man2b1, Gla, Hexa, Litaf, Npc2, Sgsh*) (Figure 6A), along with selected DAM genes involved in the transition between homeostatic state to DAM 1 and DAM 2 and seven pro‐inflammatory genes (*Nrd2, Ccr5, Psen2, Pld4, Cd180, Ets1, Cebpb*). At distance of approximately 20 μm from the deposit, we detected a distinct increase in 18 of the 28 phagocytic genes. Concurrently,  the DAM genes exhibited varying induction patterns, involving eight DAM genes of the DAM stage 1 (*B2m, Cstb, Ctsb, Ctsd, Fth1, Serinc3, Tgfbr1, Timp2*) and 3 DAM genes of the stage 2 (*Ank, Cadm1, Csf1*). Notably, at this stage we did not observe *Trem2* overexpression, which is known to be essential for the DAM stage 2 transition. Microglia located at this distance from the deposit also expressed six proinflammatory marker genes *(App, Cebpb, Nr1d1, Nr1d2, Pde2a, Tlr5*). Additionally, the genes considered homeostatic during the DAM stages showed upregulation as microglia approached the deposits (*C1qa, C1qb, C1qc, Csf1r, Cst3, Ctss, Hexb, Olfml3*).

Overall, these data suggest that phagocytic microglia dynamically regulate pro‐inflammatory and predominantly DAM genes depending on their proximity to the deposits.

In order to spatially correlate the expression of miRNA targets with the miRNA expression, we performed FISH of miR‐7a‐5p, accompanied by Aβ aggregate and Iba1 immunohistochemistry staining in slices of 6‐month old 5xFAD AD brains (Figure 6B). The majority of microglia expressing *miR‐7* *a‐5p* were located far (> 27 μm) from the Aβ deposits with a loss in expression in microglia in close vicinity to the deposits. This observation is consistent with the data obtained through spatial sequencing, where only microglia located further than 27 μm (far) from the deposits exhibited a downregulation of miR‐7 targets (Figure 6A). This concurrence supports the notion of a canonical repressive function of these miRNAs, as the expression levels of their potential targets show an inverse correlation (Figure 6A).

To analyze the phagocytic microglia signature in the previously identified DAM subtype, we performed a re‐clustering of the DAM cells in the single‐cell RNA‐seq dataset of Keren‐Shaul *et al.* ^9^ Interestingly, the Me‐X04^+^ phagocytic microglia, as defined by our miRNA target list, selectively clustered into the DAM population (Figure 6C) and was clearly separated from other immune cell types. Moreover, the expression of miRNA targets further divided the DAM population into two different clusters: phagocytic DAM (expressing phagocytic signature) and non‐phagocytic DAM (Figure 6D). Notably, our phagocytic DAM subpopulation expressed a higher level of *Trem2* in more than 75% of cells and neither of the two DAM subpopulations expressed proinflammatory genes.

To further confirm the functionality of these two subpopulations, we performed a differential expression analysis. We obtained 222 DE genes in the non‐phagocytic DAM population and 552 DE genes belonging to the phagocytic DAM. After excluding the DAM markers shared between these two populations, we found only 28 genes for non‐phagocytic DAM and 315 for phagocytic DAM microglia. IPA analysis of the phagocytic DAM DE genes revealed an increase in cell proliferation, motility, and phagocytosis along with a decrease in inflammation and cell death (Figure 6E). This result confirmed the expression of Aβ clearance‐related capacity in this distinct phagocytic subpopulation.

To enhance the clinical significance of our findings, we evaluated the expression of the key identified miRNAs in *post mortem* human cortical samples obtained from both healthy donors (*n* = 5) and AD‐diagnosed patients (*n* = 6) (Supplementary table S4). In support of our findings, we detected a significant upregulation in *miR‐29a‐3p* and *miR‐146a‐5p* in AD patients (Figure 6F), suggesting that these miRNAs may have a relevant role in the disease pathophysiology.

## 4 DISCUSSION

Over the past decade, numerous studies have established a central role for microglia in the onset and development of AD, yet the precise contribution of microglia in the disease progression and outcome remains poorly understood. Recent advances in scRNA‐seq technologies have revealed the intriguingly heterogeneity of microglia in AD. In a seminal study in 2017, Keren‐Shaul *et al.* identified a distinct microglia subtype (DAM) associated with AD progression in 5xFAD mouse model.^9^ This work delineated a transition from homeostatic microglia (Microglia 1) to DAM characterized by two stages: Stage 1 (*Trem2*‐independent) and Stage 2 (*Trem2*‐dependent). Despite the transcriptional signature indicating phagocytic potential, the functional evidence of this capability is lacking. Additionally, the regulators of microglial phagocytosis in AD remain unknown, posing challenges in developing novel therapies aimed at targeting microglia‐mediated phagocytosis.

Our study identified regulators of microglial phagocytosis and pinpointed three miRNAs (*miR‐7a‐5p*, *miR‐29a‐3p*, and *miR‐146a‐5p)* as central players in controlling microglial phagocytic capacity. Spatial transcriptomic analysis further revealed upregulation of target genes of these miRNAs in distance‐dependent manner to the Aβ deposits, accompanied by an anticorrelated expression pattern of the miRNA itself, as revealed by in situ hybridization. Furthermore, we show that the previously identified Stage 2 DAM microglia encompass a subpopulation of microglia that exhibit functional phagocytic characteristic, supported by the expression of microRNA targets involved in phagocytosis.

To investigate the transcriptional changes in phagocytic microglia in 5xFAD mice, we performed RNA‐sequencing of Me‐X04^+^ (phagocytic) and Me‐X04^‐^ (non‐phagocytic) microglia. Our data analysis revealed significant changes in microglial transcriptional profile specifically in phagocytic microglia with little dependency on the genotype (AD non‐phagocytic vs. WT non‐phagocytic). Notably, the transcription factor GATA3,  known for its role in polarization,^53^ emerged as the only transcription factor activated between TG Me‐X04^−^ and WT microglia. Interestingly, our analysis indicated that one of the annotated functions of the transcription factors regulated in phagocytic Me‐X04^+^ microglia, which obtained the highest enrichment score in our analysis, was linked to transcription of miRNA precursors.

MiRNAs are small non‐coding RNA molecules that play a crucial role in regulating gene expression by binding to specific mRNAs and inhibiting their translation into proteins or promoting their decay. As miRNAs dysregulation is associated with various diseases, including AD, these molecules show promising features as biomarkers and therapeutic tools in diseased conditions. Additionally, miRNAs can impact multiple pathways and gene targets simultaneously forming intricate regulatory circuitries that can profoundly influence treatment outcomes.

While the essential role of miRNAs in shaping microglial phenotype is well established^16^, their comprehensive role in modulating microglial phagocytosis in AD is still lacking.

In our study, the integration of mRNA and miRNA transcriptomic data revealed that only two miRNAs were induced in TG non‐phagocytic microglia, in striking contrast to the 151 identified in phagocytic TG microglia. Both of this miRNAs (*miR‐155‐5p* and *miR‐211‐5p*) were also upregulated in TG phagocytic microglia, and previous studies have suggested their upregulation to be essential in M1‐type macrophage polarization^54^, ^55^, ^56^.

The majority of the miRNAs altered in phagocytic microglia were downregulated (>70%), while their targets regulating phagocytosis‐related pathways showing upregulation, consistent with miRNAs canonical function. In vitro gain‐ and loss‐of‐function experiments showed that three of the selected and conserved miRNAs (*miR‐7a‐5p, miR‐29a‐3p, miR‐146a‐5p*) significantly reduced phagocytosis in LPS stimulated cells when overexpressed, and increased phagocytosis when inhibited.

In line with our data, *miR‐7a‐5p* levels are known to be increased within the brain of AD patients, potentially leading to inhibition of the ubiquitin‐proteasome pathway and impairing Aβ clearance.^57^, ^58^ Interestingly, we observed a downregulation of this miRNA in microglia with phagocytic activity. Similarly, the levels of *miR‐29a* were found to be elevated in plasma and cerebrospinal fluid of AD patients.^59^, ^60^ Conversely, the overall expression of *miR‐29a* expression was observed to be lower in the brain of AD patients^61^, ^62^, ^63^ and correlated with high expression of *Bace1* and thus increased Aβ production.^61^ Moreover, the induction of *miR‐29a‐3p* in peripheral blood mononuclear cells of AD patients suggested its potential role in immune cells by alleviating inflammation.^64^ Our analysis revealed an additional role of *miR‐7a‐5p* and *miR‐29a‐3p* in post‐transcriptionally regulating HIF1‐α pathway, previously suggested to regulate neuroinflammation and phagocytosis in mouse and human in the context of AD.^10^

On the other end, the available data on the expression of *miR‐146* in AD patients are conflicting,^65^, ^66^, ^67^ yet numerous studies have linked this miRNA to the regulation of the balance in the immune response in macrophages and microglia by affecting Toll‐like receptor and cytokine signaling,^68^, ^69^, ^70^ and M2 polarization.^71^, ^72^ Our mRNA‐seq analysis of BV‐2 cells overexpressing or inhibiting these miRNAs in vitro indicated that *miR‐7a‐5p* and *miR‐29a‐3p* were functional only in activated cells, suggesting that their targets may be expressed depending on the cellular activation state.

As the levels of Aβ deposits vary between cortex and hippocampi, we performed separate analysis of the spatial transcriptomic data for these areas. Interestingly, in the hippocampi, where microglia did not localize in the immediate vicinity of the Aβ deposits, we did not detect any correlation between gene expression and the vicinity of the deposits. In contrast, in the cortex, we found that microglia induced the expression of phagocytic target genes at specific distances from the Aβ deposits. This induction partially overlapped with the DAM signature genes, indicating a potential interplay between these two distinct gene patterns. In accordance with these data, in situ hybridization of miR‐7a‐5p revealed that only microglia located further than 27 μm from the deposit expressed the miRNA, while microglia in close proximity downregulated miR‐7a‐5p. This indicates an inverse correlation between miRNA and phagocytic target gene expression. These findings complement our comprehensive analysis of miRNA‐mediated regulatory networks and highlight the importance in understanding the spatial distribution and expression patterns of miRNAs in microglial responses to Aβ pathology. Our deconvolution analysis of previously published datasets revealed that DAM microglia contain a subpopulation of microglia exhibiting a phagocytic transcriptional signature, which reinforces the functional relevance of DAM microglia in Aβ clearance. We discovered that these microglia, located at approximately 20 μm from the Aβ deposits, exhibit the highest phagocytic activity, indicating a potential critical distance for microglial response to Aβ pathology. This information, together with the evidence that these miRNAs are dysregulated in AD patients, suggest that they may hold promise for potential applications in therapeutics.

In conclusion, our study enhances our understanding of the complex interplay between miRNAs, microglial phagocytosis, and Aβ pathology in AD. The elucidation of miRNA‐mediated regulatory networks provides potential therapeutic targets for Aβ clearing strategies and highlights the multifaceted role of miRNAs in shaping microglial responses in the diseased brain. As the field of miRNA‐based therapies continues to advance, while challenges remain, harnessing these regulatory molecules may pave the way for innovative therapeutic interventions and precision medicine approaches, ultimately benefiting patients with neurodegenerative disorders.

## AUTHOR CONTRIBUTIONS

Flavia Scoyni and Tarja Malm conceived and planned the study; Flavia Scoyni, Tarja Malm, Luca Giudice, Nicholas Downes provided intellectual contribution and contributed to the interpretation of the results; Flavia Scoyni, Alexandra Grubman, Paula Korhonen, Xin Yi Choo, Jose Polo, Nea Korvenlaita, Nelli‐Noora Välimäki, Petri Mäkinen and Seppo Ylä‐Herttuala contributed to the ex vivo experiments; Flavia Scoyni, Mari‐Anna Väänänen, Nea Korvenlaita and Tiia A Turunen contributed to the in vitro experiments; Luca Giudice planned and performed the bioinformatics and statistical analysis of RNA‐seq data performed by Nicholas Downes under the supervision and funding provided by Minna U Kaikkonen; Thomas B Hansen analysed public RNA‐seq datasets; Helga E de Vries and Annemieke J Rozemuller provided the *post mortem* human samples and contributed to the revision of the manuscript; Tarja Malm supervised the study; Flavia Scoyni and Tarja Malm wrote the manuscript with input from all authors. All authors provided critical feedback and helped shape the research, analysis, and manuscript.

## CONFLICTS OF INTEREST STATEMENT

The authors declare no conflicts of interest. Author disclosures are available in the supporting information.

## CONSENT STATEMENT

This study was not conducted on human subjects hence informed consent was not necessary.

The data produced in this publication have been deposited in NCBI's Gene Expression Omnibus and are accessible through GEO Series accession numbers GSE229418, GSE229417, GSE229416. The code developed to analyze the data produced in this study is deposited in Zenodo: https://doi.org/10.5281/zenodo.7802699.

## Supporting information

Supporting Information

Supporting Information

Supporting Information

Supporting Information

Supporting Information

Supporting Information

Supporting Information

Supporting Information

Supporting Information

Supporting Information

Supporting Information

Supporting Information

Supporting Information

## Data Availability

The publicly available datasets analyzed in this publication are accessible through GEO Series GSE109834 (https://www.ncbi.nlm.nih.gov/geo/query/acc.cgi?acc=GSE109834), GSE123021 (https://www.ncbi.nlm.nih.gov/geo/query/acc.cgi?acc=GSE123021), GSE75246 (https://www.ncbi.nlm.nih.gov/geo/query/acc.cgi?acc=GSE75246), GSE98401 (https://www.ncbi.nlm.nih.gov/geo/query/acc.cgi?acc=GSE98401) and GSE98969 (https://www.ncbi.nlm.nih.gov/geo/query/acc.cgi?acc=GSE98969).
