# Supplementary material for: Alzheimer's disease‐induced phagocytic microglia express a specific profile of coding and non‐coding RNAs
Source: Alzheimers Dement. 2023 Oct 12;20(2):954–74. doi: 10.1002/alz.13502 (PMC10916983; doi:10.1002/alz.13502)
Supplement: Supplementary file 1 — Supporting Information [file ALZ-20-954-s006.docx]

**Supplementary figures**


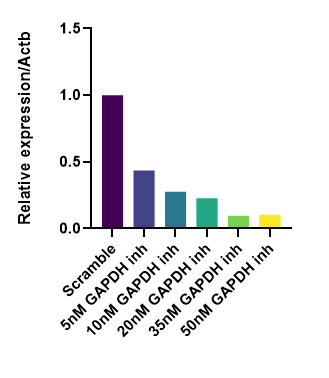


**Supplementary Figure S1. BV-2 cells mimic transfection tittering and efficiency**

Bar plot of the quantitative PCR experiment on BV-2 cells transfected with increasing amount of GAPDH inhibitor in respect of cell transfected with scramble control. The data validate the transfection efficiency obtained by FACS in **Supplementary table S1**.


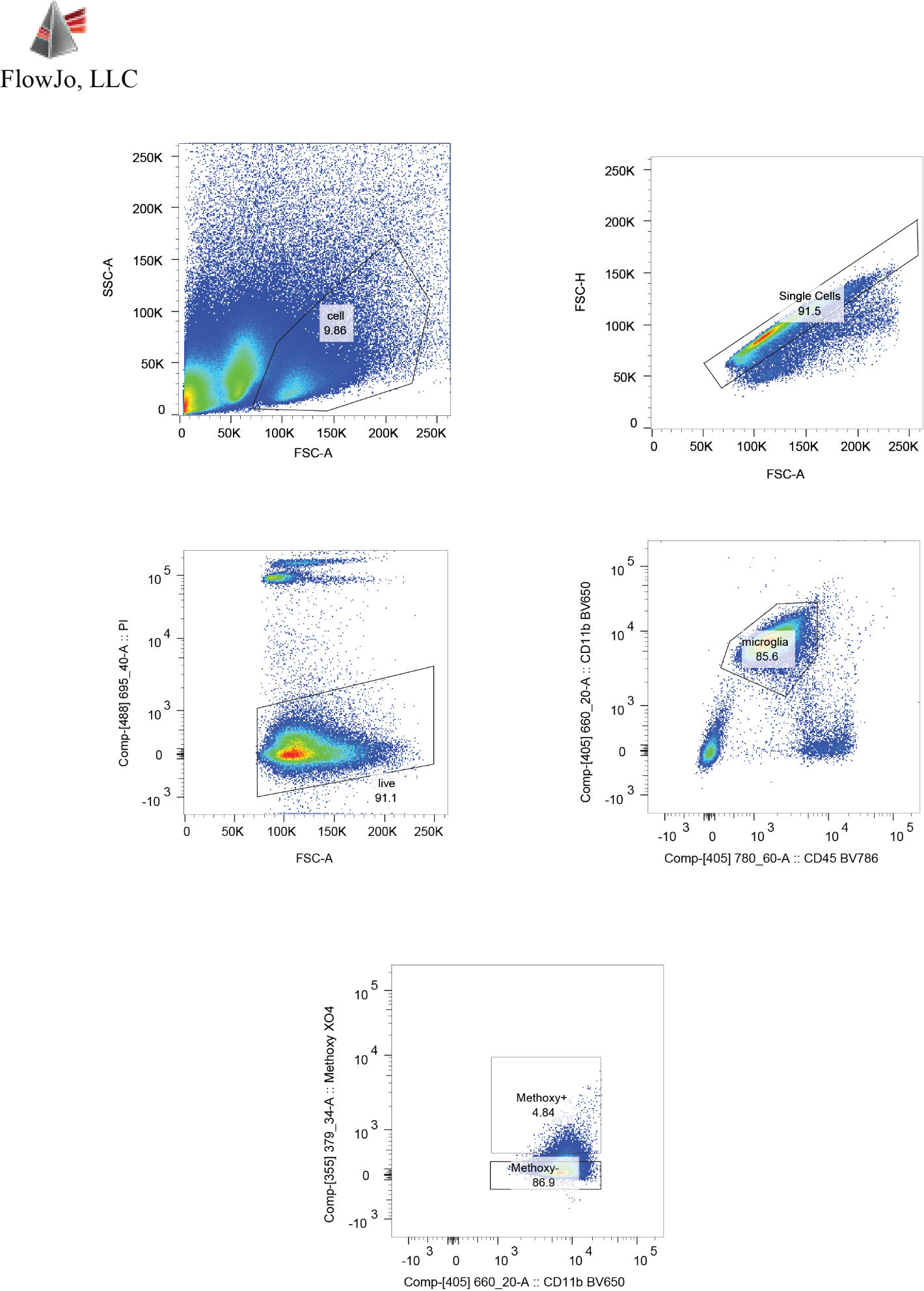


**Supplementary Figure S2. Microglia isolation gating strategy**

Cells were identified with a gate excluding debris through FSC/SSC area parameters. FSC-A/H was used to isolate singlet of cells before sorting. Propidium Iodide (PI) was used to discriminate alive cells (PI negative cells). Microglia was identified as double positive population (Cd11b, Cd45) divided for Methoxy-X04 positivity/negativity.

**
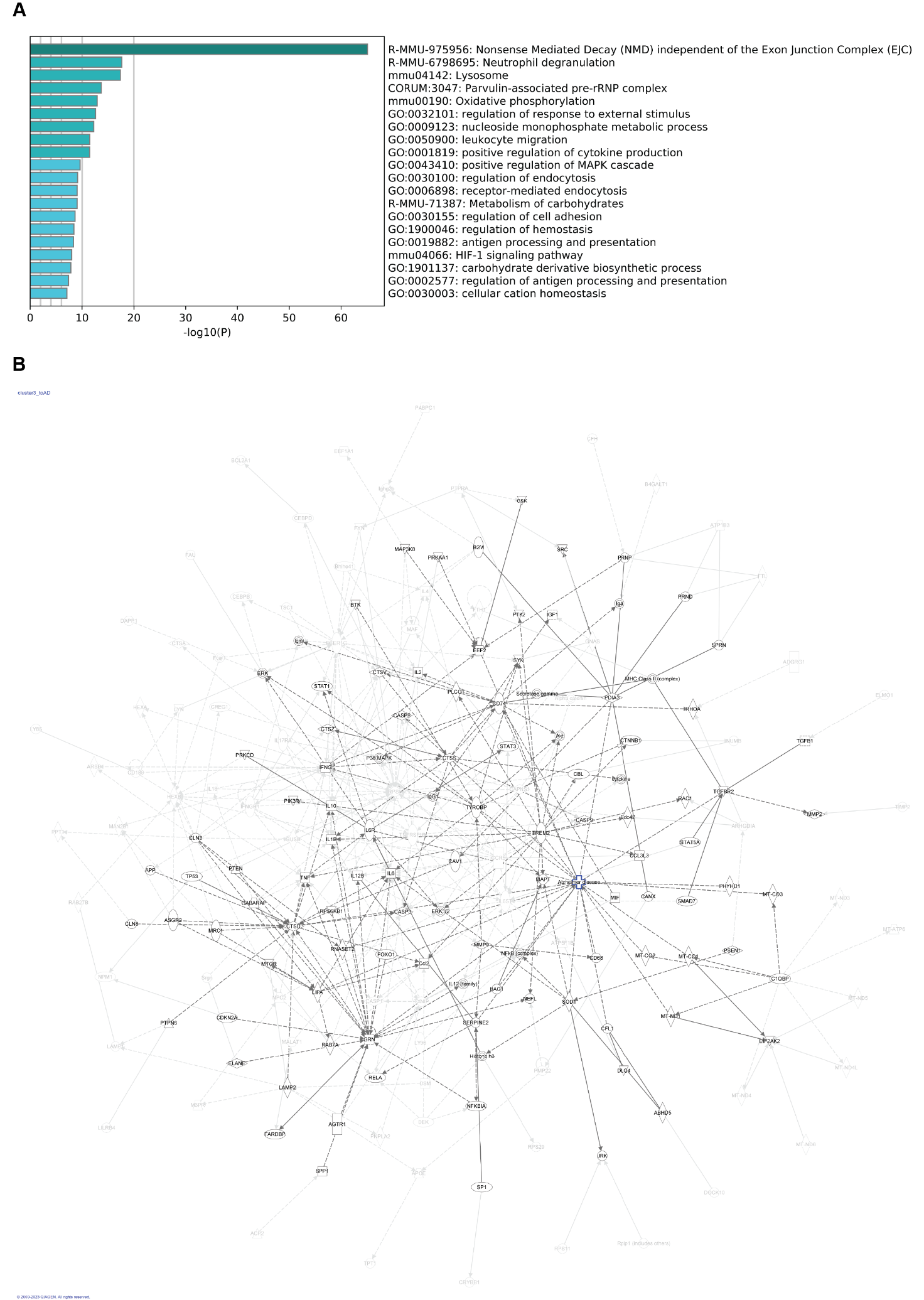
**

**Supplementary Figure S3. The opposite trending genes in Me-X04^+^ microglia identify phagocytic activity**

(**A**) Bar plot of functional enrichment analysis top 20 significant Metascape clusters performed on Cluster 3 (opposite trend genes) of the differentially expressed transcripts in phagocytic AD versus non-phagocytic microglia. (**B**) Ingenuity pathway analysis (IPA) of the genes belonging to cluster 3 (opposite trend genes) of the differentially expressed transcripts in phagocytic AD versus non-phagocytic microglia. In blue highlighted the significant strong association (probability value <0.05) of the genes belonging to this cluster and Alzheimer´s Disease (blue).


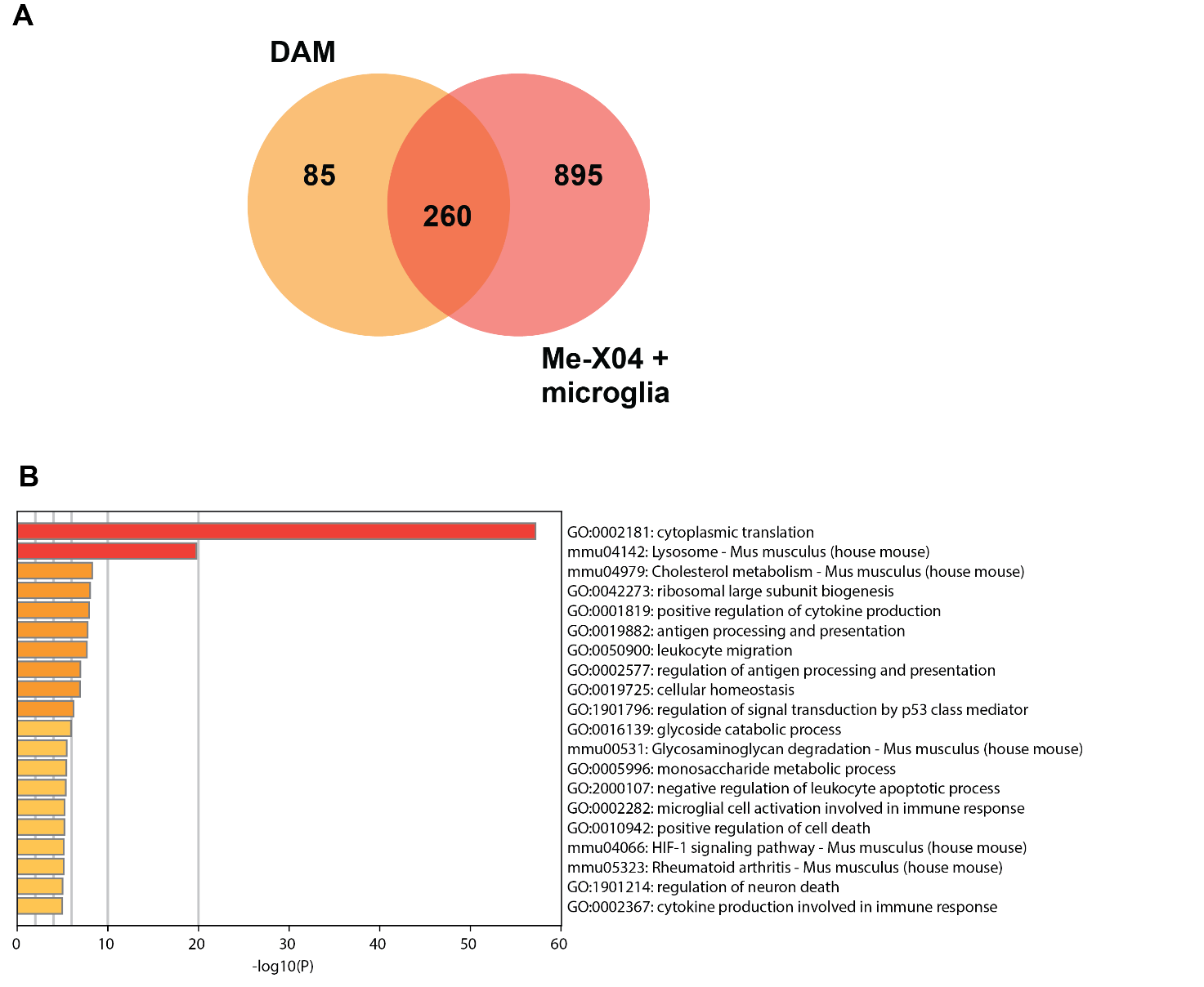


**Supplementary Figure S4. Phagocytic microglia differentially expressed genes overlap with DAM signature**

(**A**)Venn diagram of the overlap between differentially expressed genes in phagocytic microglia (pMG) from (**Supplementary table S1**) and Keren-Shaul *et al.*^9^ DAM microglia signature (DAM). For this overlap only protein coding genes were considered in both lists. (**B**) Bar plot of functional enrichment analysis top 20 significant Metascape clusters of the differentially expressed transcripts in phagocytic AD shared with DAM gene set.


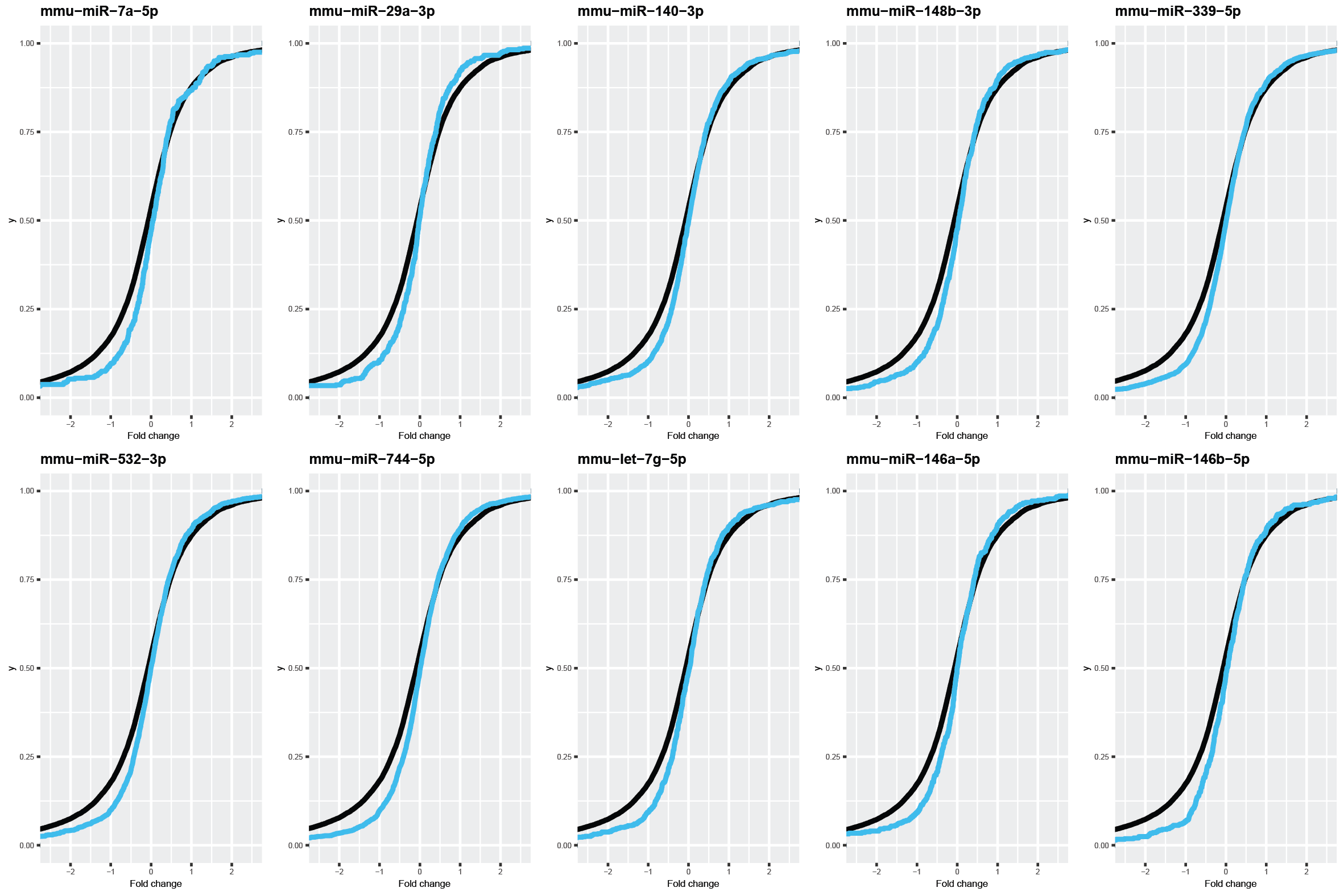


**Supplementary Figure S5. The miRNAs selected for in vitro validation affects phagocytic microglia**

Cumulative distribution functions (CDFs) of the miRWalk targets of the 9 DE miRNAs in phagocytic microglia conserved in human and expressed in BV-2 cells. Each miRNA CDF curve is obtained with the single specific miRNA targets (blue line) against all the non-targets (black line). All the results are statistically significant for derepression (n = 6 animals as biological replicates; p-value calculated with Kolmogorov-Smirnov test).
